# Modifiable risk factors for the prevention of bladder cancer: a systematic review of meta-analyses

**DOI:** 10.1007/s10654-016-0138-6

**Published:** 2016-03-21

**Authors:** Abdulmohsen H. Al-Zalabani, Kelly F. J. Stewart, Anke Wesselius, Annemie M. W. J. Schols, Maurice P. Zeegers

**Affiliations:** 1 Department of Family and Community Medicine, College of Medicine, Taibah University, P.O. Box 42317, Madinah, 41541 Saudi Arabia; 2 Department of Complex Genetics, School of Nutrition, and Translational Research in Metabolism (NUTRIM), Maastricht University Medical Centre, P. O. Box 616, 6200 MS Maastricht, The Netherlands; 3 Department of Respiratory Medicine, School of Nutrition and Translational Research in Metabolism (NUTRIM), Maastricht University Medical Centre, P. O. Box 616, 6200 MS Maastricht, The Netherlands

**Keywords:** Bladder cancer, Prevention, Risk factors, Lifestyle, Obesity, Occupation, Smoking

## Abstract

Each year, 430,000 people are diagnosed with bladder cancer. Due to the high recurrence rate of the disease, primary prevention is paramount. Therefore, we reviewed all meta-analyses on modifiable risk factors of primary bladder cancer. PubMed, Embase and Cochrane database were systematically searched for meta-analyses on modifiable risk factors published between 1995 and 2015. When appropriate, meta-analyses (MA) were combined in meta–meta-analysis (MMA). If not, the most comprehensive MA was selected based on the number of primary studies included. Probability of causation was calculated for individual factors and a subset of lifestyle factors combined. Of 1496 articles identified, 5 were combined in MMA and 21 were most comprehensive on a single risk factor. Statistically significant associations were found for current (RR 3.14) or former (RR 1.83) cigarette smoking, pipe (RR 1.9) or cigar (RR 2.3) smoking, antioxidant supplementation (RR 1.52), obesity (RR 1.10), higher physical activity levels (RR 0.86), higher body levels of selenium (RR 0.61) and vitamin D (RR 0.75), and higher intakes of: processed meat (RR 1.22), vitamin A (RR 0.82), vitamin E (RR 0.82), folate (RR 0.84), fruit (RR 0.77), vegetables (RR 0.83), citrus fruit (RR 0.85), and cruciferous vegetables (RR 0.84). Finally, three occupations with the highest risk were tobacco workers (RR 1.72), dye workers (RR 1.58), and chimney sweeps (RR 1.53). The probability of causation for individual factors ranged from 4 to 68 %. The combined probability of causation was 81.8 %. Modification of lifestyle and occupational exposures can considerably reduce the bladder cancer burden. While smoking remains one of the key risk factors, also several diet-related and occupational factors are very relevant.

## Introduction

The International Agency for Research on Cancer (IARC) estimated that worldwide 430,000 people were diagnosed with bladder cancer in 2012, with an age-standardised rate (ASR) of 5.3 per 100,000 people. Incidence of bladder cancer is particularly high in males with 77 % of the cases, ranking it the 7th highest on incidence and 9th highest on mortality. For females it is the 19th highest on incidence and 17th highest on mortality. The incidence of bladder cancer is almost 3 times higher in more developed countries compared to less developed countries (ASR of 9.5 and 3.3 per 100,000 respectively) [1].

Several genetic polymorphisms have been proposed to be associated with the development of bladder cancer. However, genetic effects can explain ‘only’ 7 % of the bladder cancer incidence in western populations [2], and therefore, risk factors related to lifestyle, environmental, and occupational exposures could potentially play an important role in the development of this disease. Over the years, multiple external risk factors have been proposed. Well-established risk factors are tobacco consumption, occupational exposure, such as to aromatic amines and Polycyclic Aromatic Hydrocarbons (PAHs), and infection with *Schistosoma hematobium* [3]. Whereas smoking is the most important risk factor in developed countries, *S. hematobium* infection accounts for the largest burden in African countries [4, 5]. Additional suggested risk factors are, amongst others, alcohol consumption, coffee intake, low fruit, and vegetable consumption, low selenium and vitamin E intake, pollution of drinking water, and several medical treatments [3, 6].

Exposure to some of the key risk factors of bladder cancer has been successfully reduced in the population. For example, the reduction in exposure to aromatic amines seemed to be directly related to a reduction in bladder cancer incidence [7]. Men are generally more exposed to occupational risk factors of bladder cancer and it is estimated that 7.1 % of the bladder cancer cases in men can be attributed to occupational factors [8]. Also, the prevalence rates of smoking have been declining significantly over the past decades, although the absolute number of smokers has been increasing due to the growing world population [9]. Because men smoke more than women, the percentage of cases attributable to smoking is higher in males than in females: 42.8 % (males) versus 25.7 % (females) in Europe, and 34.3 % (males) versus 30.1 % (females) in the United States [10]. This higher prevalence of smoking among men also explains part of the discrepancy in incidence between men and women [11]. Despite the reduction in exposure to some risk factors, significant exposure, and thus disease, remains. This means that the burden on the health care system will remain substantial especially when considering that at least 50 % of bladder cancers recur [12] and long-term monitoring of patients is required, resulting in the highest per patient lifetime costs amongst all cancers [13]. For example, in the US, the annual cost of care of bladder cancer was estimated to be $US4 billion. Therefore, primary prevention is paramount, particularly because many of the risk factors are modifiable and therefore preventable.

Prioritising control measures should be evidence-based. To this end, an accessible and comprehensive overview is needed of the modifiable risk factors in the development of bladder cancer, including estimates of public health impact. To our knowledge, no quantitative overview has been published addressing all modifiable risk factors of bladder cancer. Therefore, our aim was to give an overview of the current state of knowledge, at the highest level of evidence, with regard to modifiable lifestyle, environmental, and occupational risk factors for development of bladder cancer by performing a systematic review of meta-analyses (MAs), in combination with a series of meta–meta-analyses (MMAs).

## Methods

This systematic review was registered with PROSPERO (identification number CRD42015023411) [14]. Where relevant, the publication is written in accordance to the PRISMA guidelines [15].

### Search strategy

A systematic literature search was performed up to September 2015 in PubMed, Embase, and the Cochrane database to identify published articles of MAs and pooled analyses describing the association between lifestyle, environmental, and occupational risk factors and the development of bladder cancer. In addition, PROSPERO was searched and authors of registered but not published reviews were contacted. Finally forward and backward referencing of relevant publications was checked for additional publications.

The database was searched using the following search terms: “urinary bladder neoplasms”[MeSH Terms], “urinary bladder neoplasms”[All Fields], “bladder cancer”, combined with systematic[sb], meta-analysis[Title/Abstract], meta-analysis[Publication Type], systematic review[Text Word], pooled analysis[Text Word].

### Selection criteria for publications

A first screening was done based on the title and abstract. Articles were considered for inclusion if they were MAs or pooled analyses describing the association between modifiable risk factors and development of bladder cancer, written in English. Inclusion was limited to articles published from 1995, as during the 1990s systematic reviews and meta-analyses started to replace narrative reviews, and methods and terminology were well established by that time [16]. When the references could not be rejected with certainty, the full texts were obtained and a second screening was done. One article may report multiple MAs on different exposures.

The exposure of interest was any modifiable risk factor. ‘Modifiable’ was defined as a risk factor that is in any way reasonably modifiable, meaning that genetic and medical conditions were excluded as these are generally not modifiable. The outcome of interest was defined as incidence of bladder cancer as a binary outcome. Studies that included estimates based on prevalence or mortality were used as a proxy for incidence when estimates on incidence only were not available. Studies presenting results on genitourinary cancers without giving estimates for bladder cancer alone and studies reporting on urothelial cancer alone were excluded. Additionally, pooled risk estimates had to be presented with the accompanying 95 % confidence interval, accepted as odds ratio (OR), risk ratio (RR), hazard ratio (HR), standardised incidence ratio (SIR), or effect size (ES) if the latter consisted of a combination of the previously mentioned ratios. For the purpose of this paper, these estimates are all referred to as relative risks (RR).

### Data extraction

Data was extracted by AA and all entries were double-checked completely for errors by KS. Any disagreement was discussed between the review authors until consensus was reached. When needed, authors of publications were contacted for additional information.

For each article, the following data was extracted when available: first author, year of publication, journal, risk factor(s) studied, number of events, types of studies included, number of studies included, type of risk estimate (OR, RR, SIR), outcome variable (incidence, prevalence, mortality, or combination), and per MA (statistical procedure): the exposures that were compared, the risk estimate and the 95 % confidence interval, a measure of heterogeneity, the number of primary studies included, and a measure of publication bias.

### Selection criteria for estimates

The estimates to be included had to be based on a minimum of two primary studies. They were selected based on the following list of decisions, and in this order: (1) bladder cancer only over urothelial cancers, (2) overall estimates over sub-group estimates, (3) more adjusted estimates over less adjusted estimates, (4) smoking adjusted over other adjustments, (5) incidence over prevalence or mortality, (6) random effects model over fixed effects model, and (7) pooling of cohort studies over pooling of case–control or cross-sectional studies.

### Synthesis of results

If only one article was available on a specific risk factor, these estimates were included directly. If multiple articles were available, we selected the most comprehensive MA. This could occur in one of two ways. (1) The preferred method was to combine two MAs in a MMA to obtain an overall estimate based on the largest number of studies. However, we considered this only appropriate if the level of overlap between the primary studies included in each publication was no more than 50 %, measured as the percentage of studies of the smaller publication that overlapped with those of the larger publication. In addition, the exposure categories had to be comparable enough to be combined. (2) If, by following this rule, it was not possible to combine publications, the most comprehensive publication was included, based on the largest number of included primary studies. Basing this on the largest number of studies, rather than the largest sample size, was done because it was often not possible to determine the publication with the largest sample size due to insufficient data in the publication.

MMA was performed using a random-effects model. Heterogeneity was assessed between the included MAs using the I^2^ statistic [17]. All statistical analyses were carried out in STATA V13.0 software and *p* values of 0.05 or below, or a confidence level of 95 %, were considered statistically significant.

In order to provide a measure of public health impact, a probability of causation [18] (POC) was calculated for those risk estimates that were found to be significantly associated with the risk of bladder cancer. In addition, a combined POC [18] was calculated for the lifestyle factors that one can be exposed to at one time. Careful attention was paid to avoid overlapping exposures, such as vitamin C intake, and fruit and vegetable consumption. In that case the most all-inclusive factor was included. No such combined estimate was calculated for all occupational factors combined, as one will unlikely be exposed to multiple occupations at one time.

POC can be interpreted as the percentage of exposed cases that are attributable to the exposure (e.g. the number of cases among smokers that are attributable to smoking). In case of a protective factor this should be read as the proportion of non-exposed cases that are attributable to the fact that they are non-exposed (e.g. the number of cases among people with low fruit and vegetable intake that are attributable to the low fruit and vegetable intake).

### Role of the funding source

The funding body was not involved in the study design; in the collection, analysis, or interpretation of data; in the writing of the report; or in the decision to submit the paper for publication.

## Results

**Fig. 1:**
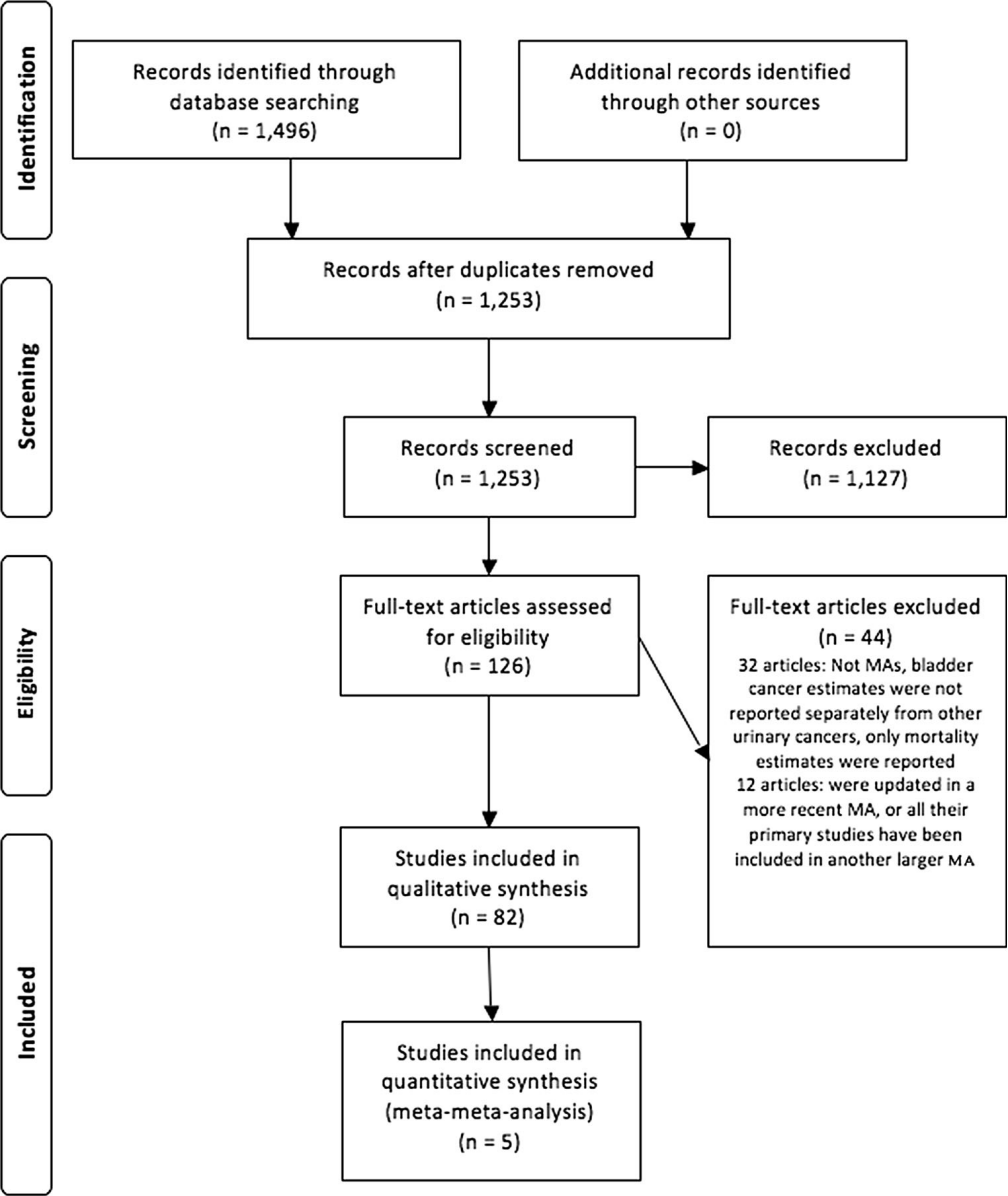
Flow diagram of literature search and selection process

**Fig. 2:**
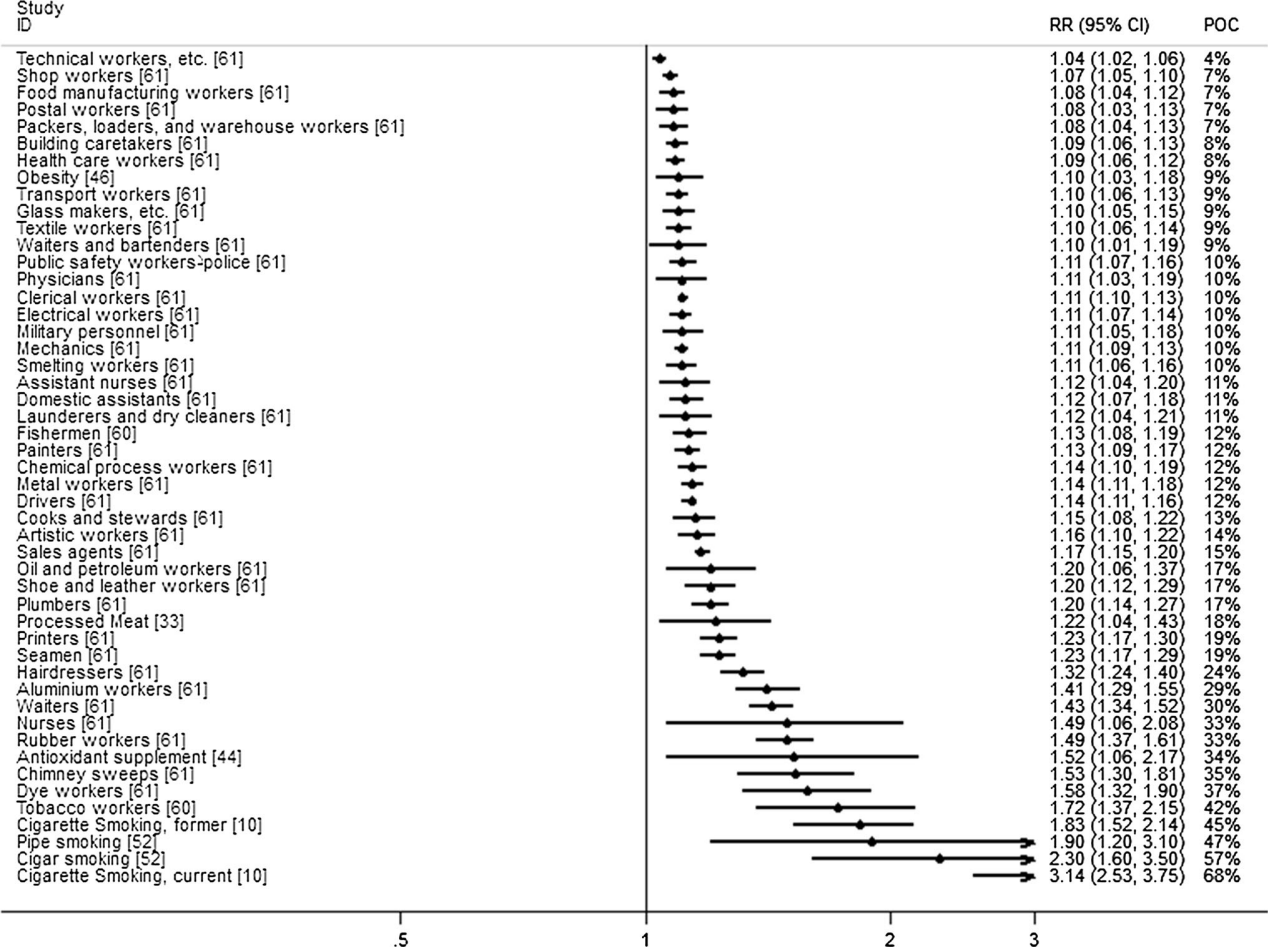
Forest plot of significantly increased risks

**Fig. 3:**
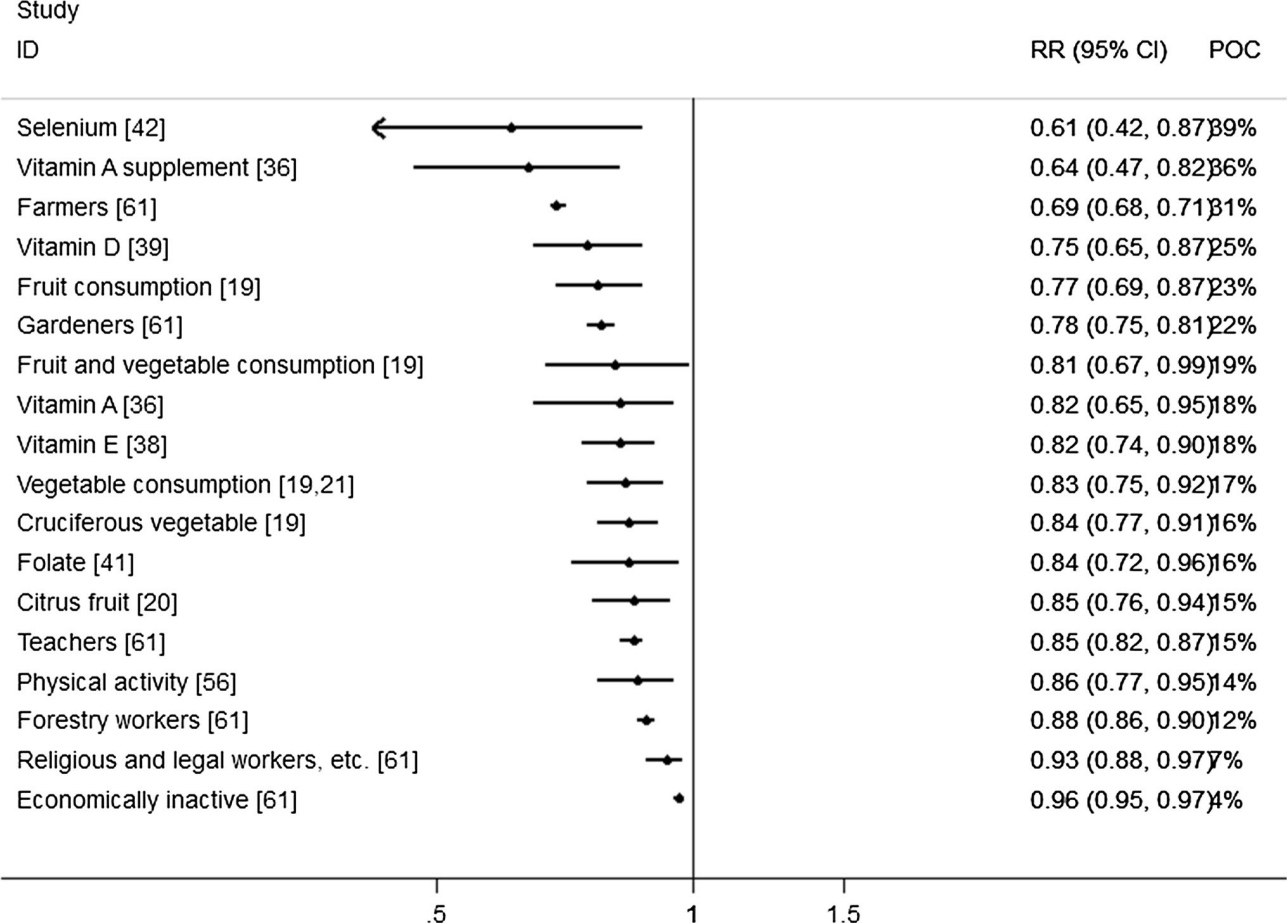
Forest plot of significantly decreased risks

Figure 1 shows the literature search and selection process. A total of 1,496 articles were identified, from which 126 articles were selected based on the title and abstract for further evaluation. Of these, 32 articles were excluded (Appendix 1) for the following reasons: not being a MA, bladder cancer estimates were not reported separately from other urinary cancers, no risk estimate reported, or only mortality estimates were reported. Furthermore, 12 articles were excluded because all included primary studies were also part of a larger MA or because an update was available. This resulted in a total of 82 eligible articles (Appendix 2). Of these 82 articles, 21 articles were selected as the most comprehensive MA for a single risk factor, and five articles were combined in two MMAs, together reporting results for 36 risk factors. The statistically significant results from all most comprehensive MAs, totalling 19 lifestyle factors and 48 occupational factors, are summarised in Table 1 and illustrated in Figs. 2 and 3, for the increased and decreased risks respectively.

**Table 1:** Modifiable risk factors with statistically significant association with bladder cancer

Risk factor	Total MAs	Total primary studies	Total BC cases in specific MA	Outcome type	Comparison	Relative risk (95 % CI)	Confidence interval	POC (%)	Combined POC (%)
*Lifestyle factors*									
Fruit and vegetable consumption [19]	1	10		NS	High versus low intake	0.81	0.67–0.99	19	^$^
Fruit consumption [19]	1	21	9867	NS	High versus low intake	0.77	0.69–0.87	23	
Citrus fruit [20]	1	14	7372	Incidence	High versus low intake	0.85	0.76–0.94	15	
Vegetable consumption [19, 21]	2 (MMA)	31 unique; 3 duplicate	8808 unique; 765 duplicate	NS	High versus low intake	0.83	0.75–0.92	17	
Cruciferous vegetable [19]	1	11	6496	NS	High versus low intake	0.84	0.77–0.91	16	
Processed meat [33]	1	11	7562	NS	High versus low intake	1.22	1.04–1.43	18	^$^
Vitamin A [36]	1	11	4990	NS	High versus low intake (dietary and supplements) and blood levels	0.82	0.65–0.95	18	
Vitamin A supplement [36]	1	5	1403	Incidence	Supplementation versus placebo or no supplementation	0.64	0.47–0.82	36	
Vitamin D [39]	1	5	2238	Incidence/mortality	High versus low serum level	0.75	0.65–0.87	25	
Vitamin E [38]	1	15	5224	Incidence	High versus low intake	0.82	0.74–0.90	18	
Folate [41]	1	13	6280	Incidence	High versus low intake	0.84	0.72–0.96	16	
Selenium [42]	1	7	1014	Incidence	High versus low serum or toenail level	0.61	0.42–0.87	39	
Antioxidant supplement [44]	1	4	NR	Incidence	Supplementation versus placebo or no supplementation	1.52	1.06–2.17	34	
Obesity [46]	1	12		NS	Obese versus normal body weight	1.10	1.03–1.18	9	
Cigarette smoking [10]	1	13	9129	Incidence	Current cigarette smokers versus never smokers	3.14	2.53–3.75	68	^$^
	1	12	8659	Incidence	Former cigarette smokers versus never smokers	1.83	1.52–2.14	45	
Pipe smoking [52]	1	6	34	Incidence	Pipe only smokers versus never smokers	1.90	1.2–3.1	47	
Cigar smoking [52]	1	6	52	Incidence	Cigar only smokers versus never smokers	2.30	1.6–3.5	57	
Physical activity [56]	1	7	NR	Incidence	High versus low	0.86	0.77–0.95	14	^$^
Combined POC									81.8
*Occupational factors* [61]									
Tobacco workers	1	3	87	Incidence	Versus any other occupation	1.72	1.37–2.15	42	
Dye workers	1	21	344	Incidence	Versus any other occupation	1.58	1.32–1.90	37	
Chimney sweeps	1	4	146	Incidence	Versus any other occupation	1.53	1.30–1.81	35	
Nurses	1	13	1195	Incidence	Versus any other occupation	1.49	1.06–2.08	33	
Rubber workers	1	45	1263	Incidence	Versus any other occupation	1.49	1.37–1.61	33	
Waiters	1	9	1146	Incidence	Versus any other occupation	1.43	1.34–1.52	30	
Aluminium workers	1	21	977	Incidence	Versus any other occupation	1.41	1.29–1.55	29	
Hairdressers	1	47	1445	Incidence	Versus any other occupation	1.32	1.24–1.40	24	
Printers	1	42	1724	Incidence	Versus any other occupation	1.23	1.17–1.30	19	
Seamen	1	10	1791	Incidence	Versus any other occupation	1.23	1.17–1.29	19	
Oil and petroleum workers	1	17	637	Incidence	Versus any other occupation	1.20	1.06–1.37	17	
Shoe and leather workers	1	39	1091	Incidence	Versus any other occupation	1.20	1.12–1.29	17	
Plumbers	1	8	1418	Incidence	Versus any other occupation	1.20	1.14–1.27	17	
Sales agents	1	91	11923	Incidence	Versus any other occupation	1.17	1.15–1.20	15	
Artistic workers	1	34	1678	Incidence	Versus any other occupation	1.16	1.10–1.22	14	
Cooks and stewards	1	15	1117	Incidence	Versus any other occupation	1.15	1.08–1.22	13	
Chemical process workers	1	79	3712	Incidence	Versus any other occupation	1.14	1.10–1.19	12	
Metal workers	1	62	5461	Incidence	Versus any other occupation	1.14	1.11–1.18	12	
Drivers	1	57	10396	Incidence	Versus any other occupation	1.14	1.11–1.16	12	
Fishermen	1	7	1525	Incidence	Versus any other occupation	1.13	1.08–1.19	12	
Painters	1	43	3472	Incidence	Versus any other occupation	1.13	1.09–1.17	12	
Assistant nurses	1	2	812	Incidence	Versus any other occupation	1.12	1.04–1.20	11	
Domestic assistants	1	35	1776	Incidence	Versus any other occupation	1.12	1.07–1.18	11	
Launderers and dry cleaners	1	19	767	Incidence	Versus any other occupation	1.12	1.04–1.21	11	
Public safety workers–police	1	30	2382	Incidence	Versus any other occupation	1.11	1.07–1.16	10	
Physicians	1	10	858	Incidence	Versus any other occupation	1.11	1.03–1.19	10	
Clerical workers	1	148	21109	Incidence	Versus any other occupation	1.11	1.10–1.13	10	
Electrical workers	1	45	5314	Incidence	Versus any other occupation	1.11	1.07–1.14	10	
Military personnel	1	14	2091	Incidence	Versus any other occupation	1.11	1.05–1.18	10	
Mechanics	1	127	67195	Incidence	Versus any other occupation	1.11	1.09–1.13	10	
Smelting workers	1	24	2319	Incidence	Versus any other occupation	1.11	1.06–1.16	10	
Transport workers	1	35	5243	Incidence	Versus any other occupation	1.10	1.06–1.13	9	
Glass makers, etc.	1	29	2219	Incidence	Versus any other occupation	1.10	1.05–1.15	9	
Textile workers	1	79	3822	Incidence	Versus any other occupation	1.10	1.06–1.14	9	
Waiters and bartenders	1	34	1179	Incidence	Versus any other occupation	1.10	1.01–1.19	9	
Building caretakers	1	4	3246	Incidence	Versus any other occupation	1.09	1.06–1.13	8	
Health care workers	1	20	1072	Incidence	Versus any other occupation	1.09	1.06–1.12	8	
Food manufacturing workers	1	33	3559	Incidence	Versus any other occupation	1.08	1.04–1.12	7	
Postal workers	1	4	1791	Incidence	Versus any other occupation	1.08	1.03–1.13	7	
Packers, loaders, and warehouse workers	1	25	3586	Incidence	Versus any other occupation	1.08	1.04–1.13	7	
Shop workers	1	4	6068	Incidence	Versus any other occupation	1.07	1.05–1.10	7	
Technical workers, etc.	1	37	11579	Incidence	Versus any other occupation	1.04	1.02–1.06	4	
Economically inactive	1	2	23436	Incidence	Versus any other occupation	0.96	0.95–0.97	4	
Religious and legal workers, etc.	1	6	1754	Incidence	Versus any other occupation	0.93	0.88–0.97	7	
Forestry workers	1	53	10865	Incidence	Versus any other occupation	0.88	0.86–0.90	12	
Teachers	1	30	3884	Incidence	Versus any other occupation	0.85	0.82–0.87	15	
Gardeners	1	10	3308	Incidence	Versus any other occupation	0.78	0.75–0.81	22	
Farmers	1	73	16607	Incidence	Versus any other occupation	0.69	0.68–0.71	31	

*BC* bladder cancer, *MA* meta-analysis, *MMA* meta-meta-analysis, *NS* not specified, *POC* probability of causation

^$^Risk factors and protective factors that have been included in the combined POC. The POC can be interpreted as the proportion of exposed cases that are attributable to the exposure

### Lifestyle factors

Four general topics emerged from the literature search with regard to lifestyle: dietary factors, consumption of tobacco, alcohol consumption, and physical activity.

#### Dietary factors

##### Fruit and vegetable

The MA by Yao et al. [19] was selected as the most comprehensive publication for overall fruit and vegetable intake and reported significant protective effect on bladder cancer (RR 0.81; 95 % CI 0.67–0.99; I^2^ = 58.4 %; n = 10).

##### Fruit only

MA by Yao et al. [19] was selected as most comprehensive for consumption of overall fruit and the MA by Liang et al. [20] for intake of citrus fruit (e.g. oranges, lemons, limes, and grapefruits;). Overall fruit was associated with a reduced risk of bladder cancer (RR 0.77; 95 % CI 0.69–0.87; I^2^ = 54.9 %; n = 27) as were citrus fruit (highest vs. lowest intake: RR 0.85; 95 % CI 0.76–0.94; I^2^ = 72.1 %; n = 14). Yao et al. [19] reported significant publication bias for overall and citrus fruit intake.

##### Vegetables only

By pooling results from Yao et al. [19] and Steinmaus et al. [21] in a MMA, the most comprehensive estimate could be obtained for overall vegetable consumption (n = 31 unique and n = 3 duplicate primary studies). The MA by Yao et al. [19] was selected as the most comprehensive publication for cruciferous vegetables. Consumption of overall vegetables (RR 0.83; 95 % CI 0.75–0.92; I^2^ = 0.0 %; n = 21 + 8) and cruciferous vegetables specifically (RR 0.84; 95 % CI 0.77–0.91; n = 11), resulted in a reduced risk of bladder cancer.

#### Total fluid

Bai et al. [22] was the most comprehensive estimate and found no statistically significant association between total fluid intake and bladder cancer (RR 1.12; 95 % CI 0.94–1.33; I^2^ = 82.8 %; n = 14).

#### Tea

The MA by Qin et al. [23] was selected as most comprehensive and they found a significant protective effect for black tea consumption (RR 0.79; 95 % CI 0.59–0.99; I^2^ = 33.1 %; n = 7) but not for overall and green tea consumption.

#### Coffee

Six MAs [24–28] reported on the association between coffee consumption and bladder cancer risk, of which Villanueva et al. [27], Yu et al. [26], and Wu et al. [29] could be combined into a single estimate to provide the most comprehensive estimate (n = 38 unique and n = 3 duplicate primary studies). No statistically significant association was observed, with substantial heterogeneity (RR 1.12; 95 % CI 0.80–1.44; I^2^ = 94.2 %; n = 18 + 9 + 6).

#### Sweetened carbonated beverages

Only one MA by Boyle et al. [30] was identified, reporting on the association between sweetened carbonated beverage consumption and risk of bladder cancer. No statistically significant association was found (RR 1.13; 95 % CI 0.89–1.45; n = 5).

#### Milk and dairy products

As reported by Mao et al. [31], which was selected as most comprehensive, overall milk, skim milk, and fermented milk were associated with a significantly reduced incidence of bladder cancer (milk: RR 0.84; 95 % CI 0.72–0.97; I^2^ = 70.1 %; n = 16; skim milk: RR 0.47; 95 % CI 0.18–0.79; I^2^ = 0; n = 2; fermented milk: RR 0.69; 95 % CI 0.47–0.91; I^2^ = 62.5; n = 5). In contrast, consumption of whole milk was associated with a significantly increased risk of bladder cancer (RR 2.23; 95 % CI 1.45–3.00; I^2^ = 0; n = 2).

#### Fish

The MA by Li et al. [32] was the only MA that studied the association between fish consumption and bladder cancer and reported no statistically significant association (RR 0.86; 95 % CI 0.61–1.12).

#### Meat

The MA by Li et al. [33] was selected as most comprehensive and they found an increased risk of bladder cancer associated with processed meat (RR 1.22; 95 % CI 1.04–1.43; n = 11) but not with red meat (RR 1.15; 95 % CI 0.97–1.36; n = 14).

#### Alcohol

The MA by Pelucchi et al. [34] was considered most comprehensive and they found no statistically significant association for heavy drinkers (≥ 3 drinks (≥ 37.5 g)/day; RR 0.97; 95 % CI 0.72–1.31; n = 7) or moderate drinkers (<3 drinks/day; RR 0.98; 95 % CI 0.89–1.07; n = 15).

#### Egg

The MA by Li et al. [35] was the only MA identified in which no significant association was detected for overall egg consumption (RR 1.11; 95 % CI 0.73–1.69; n = 6). However, when looking at cooking methods, an increased risk of bladder cancer was found for fried egg (RR 2.04; 95 % CI 1.41–2.95; n = 2) but not for boiled egg (RR 1.25; 95 % CI 0.82–1.91; n = 2).

#### Vitamin A: body levels, intake, and supplementation

The MA by Tang et al. [36] was selected as most comprehensive MA for both overall intake and body levels of vitamin A, and vitamin A supplementation. They found a significantly lower risk of bladder cancer among individuals with higher dietary intake or higher blood levels of vitamin A (RR 0.82; 95 % CI 0.65–0.95; I^2^ = 46.3 %; n = 11). When looking only at intake of vitamin A, the association was significantly protective for vitamin A supplementation, consisting of beta-carotene, and/or retinol supplementation (highest vs. lowest: RR 0.64; 95 % CI 0.47–0.82; I^2^ = 0 %) but not for dietary intake (highest vs. lowest: RR 0.90; 95 % CI 0.80–1.01; I^2^ = 0 %).

Jeon et al. [37] investigated the effect of only beta-carotene supplementation (so not retinol), compared to no supplementation, and found an increased risk of bladder cancer (RR 1.52; 95 % CI 1.03–2.24; I^2^ = 0.0 %; n = 2).

#### Vitamin C

The MA by Wang et al. [38] was the only MA studying the association between vitamin C intake and bladder cancer. Combining vitamin C intake from diet and supplementation, they found a marginally significantly decreased risk of bladder cancer (RR 0.90; 95 % CI 0.79–1.00; I^2^ = 43.7 %; n = 20).

#### Vitamin D

The MA by Liao et al. [39] investigated only the role of vitamin D serum level. They found that the risk of bladder cancer was decreased among individuals with the highest levels of serum vitamin D (RR 0.75; 95 % CI 0.65–0.87; n = 5). The MA by Chen et al. [40] reported on the role of vitamin D intake from diet and supplements. Combining vitamin D intake from diet and supplementation, they found no significantly altered risk of bladder cancer (RR 0.92; 95 % CI 0.66–1.28; n = 3).

#### Vitamin E

The MA by Wang et al. [38] was selected as the most comprehensive MA and reported significantly reduced risk of bladder cancer for vitamin E intake (RR 0.82; 95 % CI 0.74–0.90; I^2^ = 0 %; n = 15).

#### Folate

The association between folate intake and risk of bladder cancer was studied only in a MA by He et al. [41]. They found a significantly reduced risk of bladder cancer (RR 0.84; 95 % CI 0.72–0.96; I^2^ = 28.9 %; n = 13). The association remained significantly reduced when pooling studies on dietary folate only (RR 0.82; 95 % CI 0.65–0.99; I^2^ = 57.9 %; n = 9), but not on supplemental folate only (RR 0.91; 95 % CI 0.58–1.25; I^2^ = 62.6 %; n = 3).

#### Selenium: body levels and supplementation

Two MAs [42, 43] were identified studying the association between selenium levels in the body, measured in serum or toenail, and bladder cancer. Amaral et al. [42] was selected as the most comprehensive MA. They found that the risk of bladder cancer was significantly lower among individuals with the highest selenium levels in the body (RR 0.61; 95 % CI 0.42–0.87; n = 7).

Selenium supplementation was studied in one MA by Vinceti et al. [43]. They meta-analysed two RCTs and found no statistically significant association (RR 1.14; 95 % CI 0.81–1.61; n = 2).

#### Antioxidant supplementation

One MA by Myung et al. [44] reported on multiple types of antioxidant supplementation together, studied in RCTs. They found that antioxidant supplementation significantly increased the risk of bladder cancer (RR 1.52; 95 % CI 1.06–2.17; I^2^ = 0.0 %; n = 4).

#### Dietary acrylamide

The association between dietary acrylamide intake and bladder cancer was studied in one MA, authored by Pelucchi et al. [45] They reported no statistically significant association (RR 0.93; 95 % CI 0.78–1.11; n = 3).

#### Obesity

Two MAs [46, 47] reported on the association between body weight and bladder cancer. The MA by Sun et al. [46] was selected as most comprehensive. They found an increased risk of bladder cancer among obese individuals (BMI ≥ 30; RR 1.10; 95 % CI 1.03–1.18; I^2^ = 8.8 %; n = 12) but not among pre-obese individuals (BMI 25.00–29.99; RR 1.07; 95 % CI 0.99–1.16; I^2^ = 46.1 %; n = 13).

#### Consumption of tobacco

##### Active smoking

Eight MAs [10, 11, 48–53] were identified on the effect of active smoking of tobacco on the risk of bladder cancer. With regard to cigarette smoking, Cumberbatch et al. [53] and Van Osch et al. [10] both included almost the same number of primary studies: n = 90 versus n = 89 respectively. Although theoretically this meant that the paper by Cumberbatch et al. would be most comprehensive, after discussion between the authors, the paper by Van Osch et al. was selected as most comprehensive as they reported dose–response analyses on duration, intensity, and time since cessation of smoking. Because smoking was already known to be the most important modifiable risk factor of bladder cancer, the dose–response variables were considered valuable additional information. It must be noted that calculated risks do not differ much between both MAs (Appendix 2).

Van Osch et al. [10] reported a significantly increased risk of more than 3 times in current smokers (RR 3.14; 95 % CI 2.53–3.75) and almost 2 times in former smokers (RR 1.83; 95 % CI 1.52–2.14). Their dose–response associations showed a positive trend with increasing intensity of smoking and number of pack years, reaching a plateau from about 15 cigarettes/day and 50 pack-years. Increasing duration since cessation of smoking resulted in a reducing risk, although former smokers remained at a 50 % increased risk even after more than 20 years of cessation.

Hemelt et al. [11] also reported on the risk among smokers of any type of tobacco. They found an increased incidence of bladder cancer among current smokers (RR 3.35; 95 % CI 2.90–3.88; n = 11) as well as among ever-smokers (RR 2.25; 95 % CI 1.96–2.59; n = 15).

Two MAs [52, 53] additionally reported on the effect of pure cigar and pure pipe smoking. Pitard et al. [52] was considered most comprehensive and they reported a significantly increased risk of bladder cancer among both pipe smokers (RR 1.9; 95 % CI 1.2–3.1; n = 6) and cigar smokers (RR 2.3; 95 % CI 1.6–3.5; n = 6).

##### Passive smoking

Van Hemelrijck et al. [54] reported on the association between passive smoking and bladder cancer. They found no statistically significant association in individuals exposed to passive smoking (RR 0.99; 95 % CI 0.86–1.14; I^2^ = 35.6 %; n = 8) compared to never-smoking individuals with no exposure to passive smoking.

##### Smokeless tobacco

Use of smokeless tobacco, including chewing tobacco, oral snuff, and unspecified smokeless tobacco, was studied in two MAs [53, 55]. Lee et al. [55] was considered most comprehensive. No statistically significant association was found with development of bladder cancer (RR 0.95; 95 % CI 0.71–1.29; n = 9).

#### Physical activity

Keimling et al. [56] wrote the only MA on the association between physical activity and bladder cancer. They found a statistically significant protective effect on the bladder cancer risk among individuals with the highest levels of physical activity (RR 0.86; 95 % CI 0.77–0.95; n = 7).

#### Personal hair dye use

Turati et al. [57] updated previous MAs [58–60] to investigate the association between personal use of hair dye and bladder cancer. They found no statistically significant increased risk among users of personal hair dye (smoking adjusted RR 0.94; 95 % CI 0.82–1.08; n = 12).

### Occupational factors

Multiple MAs were identified on the association between occupational factors and bladder cancer. Most MAs reported on a single occupation or occupational group, or a single specific occupational exposure (Appendix 2). Moreover, four MAs [61–64] reported on a large number of occupational categories. Cumberbatch et al. [61] and Reulen et al. [62] authored two large MAs on different occupations based on 217 and 130 different studies respectively, whereas Kogevinas et al. [63] and ‘t Mannetje et al. [64] both did a pooled analysis of eleven case–control studies conducted in European countries reporting results for men and women, respectively. The paper by Cumberbatch et al. was considered the most comprehensive for all identified occupations based on the number of studies included. They found the following occupations to have a statistically significant increased risk of more than 20 %: tobacco workers, dye workers, chimney sweeps, nurses, rubber workers, waiters, aluminium workers, hairdressers, printers, seamen, oil and petroleum workers, shoe and leather workers, and plumbers. Protective effects were found for farmers, gardeners, teachers, forestry workers, religious and legal workers, and economically inactive workers. Statistically significant associations reported by Cumberbatch et al. are listed in Table 1. All other associations are summarized in Appendix 3 along with findings by Reulen et al. [62], Kogevinas et al. [63], and ‘t Mannetje et al. [64].

### Probability of causation

The estimates for probability of causation (POC), displayed in percentages, are presented in Table 1. The percentages ranged from 9 to 68 % among the lifestyle factors, and between 4 and 42 % for the occupational exposures. The four highest POCs are found among the smoking estimates: 68 % of the bladder cancer incidence among cigarette smokers, 57 % among cigar smokers, 47 % among pipe smokers and 45 % among former cigarette smokers can be attributed to this habit.

A POC was calculated for four factors combined, which were considered sufficiently independent: total fruit and vegetable consumption, processed meat consumption, smoking, and physical activity. To avoid overlap, the only estimate included for smoking was for cigarette smoking as most smokers are (also) cigarette smokers (78.7 %) and only 4.5 % was found to be pure pipe and/or cigar smoker [52]. The combined POC showed that up to 81.8 % of the bladder cancer cases, among those with non-optimal lifestyle behaviours, could be prevented through lifestyle modifications.

## Discussion

In total, 12 lifestyle factors and 48 occupational factors emerged that significantly increased or decreased the risk of bladder cancer. Such numerous modifiable risk factors present an opportunity and great potential for prevention. Below, the significantly associated risk factors will be discussed.

### Smoking

The most important risk factor proved to be smoking, particularly the risk was highest for current smokers of cigarettes (RR 3.14), [10] but also for smokers of cigars only (RR 2.3) and pipes only (RR 1.9) [52]. An interesting finding was that a plateau was reached at about 15 cigarettes/day, meaning that heavier smoking does not carry a much higher risk than less heavy smoking [10]. Although the risk slowly decreased with longer cessation of cigarette smoking, the RR was still 50 % increased 20 years of cessation.

Characteristics of the tobacco smoked may influence this association. In previous studies, individuals smoking black tobacco had a higher risk compared to smokers of blond tobacco, due to the higher concentrations of carcinogens in black tobacco [65–71]. Also, inhalation of the tobacco smoke into the lungs and throat resulted in a higher risk compared to inhalation only into the mouth, [49, 65, 71–76] although not all evidence supports this [77–80].

Tobacco smoke contains numerous carcinogens that contribute to the initiation and promotion of tumour development. These chemicals are renally excreted, making it directly toxic to the human urinary bladder. During metabolisation of these compounds, DNA-adducts are formed, leading to permanent genetic mutations. If this occurs in an oncogene or tumour suppressor gene, it may result in uncontrolled growth and eventually cancer.

Genetic mutations, such as NAT2 slow acetylation and GSTM null genotypes, have previously been shown to be associated with increased susceptibility to cancer [3]. The enzymes for which the genes code play a role in the detoxification pathways of i.a. aromatic amines and PAHs. These mutations do not intrinsically cause bladder cancer but do increase susceptibility when exposed to tobacco smoke or other sources of exposure [81, 82].

### Dietary factors

Because the metabolites of many food groups are excreted by the urinary tract, dietary factors have often been suggested and researched as risk factors of bladder cancer [83]. Several different nutritional factors have been identified, showing both increased and decreased risks of bladder cancer.

#### Fruit, vegetables, and antioxidants

One key group of foods that was associated with a lower risk of bladder cancer were fruits and vegetables. Besides these groups as a whole, intake of both citrus fruit and cruciferous vegetables were researched. The effect is suggested to be due to the vitamins, minerals, and phytochemicals that fruit and vegetables contain, which have antioxidant properties [84, 85]. Antioxidants may protect against cancer through inhibition of oxidation of DNA, controlling of cell proliferation and apoptosis [85], and facilitation of metabolisation of carcinogenic compounds into less toxic substances, although the association may be dose-dependent [86].

It should be noted that significant publication bias was found in the MA on overall and citrus fruit consumption [19]. Because small studies with negative results tend not to be published, the effect size found in the MAs on these risk factors may actually be attenuated.

The MAs included in our review that studied vitamins separately, came to supporting conclusions. For all but vitamin C, higher intakes were associated with a significantly reduced risk of bladder cancer, with effects on −39 % for selenium, −18 % for vitamin A and E, and −16 % for folate.

Vitamin A intake was found to be negatively associated with bladder cancer when data on dietary intake, supplementation, and blood levels were combined. However, when studied by intake source separately, the protective effect was only found for vitamin A supplementation but not for dietary intake [36]. Furthermore results on supplementation were not consistent as Jeon et al. [37], found a significantly increased risk of bladder cancer of 52 % among participants taking supplementation in experimental studies. These conflicting results may be the result of a dose-dependent relationship, showing intake to be protective at low doses and harmful at high doses. Another explanation may be the different study design, in which the reduced risk found in observational studies may actually be the effect of an overall healthier lifestyle, as those individuals who choose to use nutritional supplementation tend to have a healthier lifestyle overall [87], Vitamin A plays an important role in cell proliferation and differentiation [88], and carotenoids can directly affect carcinogenesis through their antioxidant properties, or indirectly after transformation of provitamin A carotenoids into vitamin A [89, 90].

Vitamin E consists of a group of lipid soluble molecules, including four tocopherols and four tocotrienols. Specifically γ-tocopherol, δ-tocopherol, and tocotrienols inhibit several inflammatory pathways, [91] which is important because chronic inflammation or chronic infection influences development and progression of cancer, [92] In addition, vitamin E is a potent lipid-soluble antioxidant, inhibiting lipid peroxidation and thereby protecting cell membranes from peroxidative damage [93].

Although higher levels of selenium protected against bladder cancer, causation could not be confirmed through supplementation intervention [42], The specific mechanism of action of selenium in carcinogenesis is not yet well understood. Several theories have been suggested, including selenoproteins serving as antioxidants and metabolites of selenium involved in redox cycling, modification of protein thiols, and methionine mimicry [94].

However, despite these suggested protective properties of antioxidants, analysis of multiple types of antioxidant supplementation together, showed a 52 % increased risk of bladder cancer [44]. An explanation for this discrepancy may be that it is not one antioxidant alone that has cancer-preventative properties, but rather the interaction between multiple antioxidants and other phytochemicals, such as it occurs with consumption of a diet rich in fruit and vegetables.

#### Other nutrients and food groups

Because consumption of processed meat but not red meat was associated with bladder cancer, the effect may result from different forms of mechanical, chemical, and enzymatic treatment that processed meat has been exposed to. One hypothesis suggests that nitrite, which is used as a colour and flavour preservative in processed meat, combines with secondary amines from proteins to form nitrosamines that are found to be carcinogenic to, amongst other organs, the bladder [95]. Although red and processed meat are often studied in relation to disease development, particularly cancer, the exact mechanism of action is still unclear and is likely to be a combination of factors present in or associated with meat consumption [96, 97].

The protective effect of folate, a vitamin predominantly found in vegetables, is suggested to be through its role in DNA synthesis, repair, and methylation [98]. In previous research, low levels of folate were associated with hypomethylation of DNA resulting in dysregulation of proto-oncogenes and tumour suppressor genes, and therefore an increased risk of cancer [99]. However, the association is not as straightforward as may be expected. Research suggests that timing and dose of folate plays an important role in its effect on cancer development [100]. Because folate plays a role in de-novo synthesis of nucleotides, it will support healthy tissue and prevent tumour development. However, once pre-neoplastic lesions have been established, it will also support these rapidly proliferating tissues resulting in rapid tumour progression.

In line with previous findings for colorectal cancer [101] and breast cancer, [102] higher serum vitamin D was found to be protective against bladder cancer. Calcitriol, the potent hormone produced in the body from vitamin D, plays a role in multiple stages of cancer development, through both genomic and non-genomic pathways [103]. No association was found for dietary and supplemental intake of vitamin D, which may result from dietary intakes not adequately reflecting calcitriol available to the body as it is also endogenously formed in response to UV-light.

Previous reviews have also mentioned other dietary factors increasing the risk of bladder cancer, among which higher intake of soy, fat, barbecued meats, and artificial sweeteners [84, 104]. However, no MAs were identified on these factors so these factors have not been included in our overview.

### Fluid intake and water contaminants

Whereas increased intake of fluids dilutes urine and increases micturition, leading to a reduced exposure of the bladder to carcinogens, it may also increase exposure when the fluid itself contains carcinogens [3]. For example, increased risk of bladder cancer was found with water chlorination [105, 106] and arsenic contamination [107, 108]. However, no significant association was found for either all fluid intake or water specifically [22]. Together, this is in line with the suggested dual effect of increased fluid intake on bladder cancer risk.

### Physical activity

Physical activity was found to have a small but significant protective effect against bladder cancer. The effect found was not modified by BMI or smoking, as the authors explain in their MA [56]. Although the exact mechanism through which physical activity protects against cancer is not yet known, several mechanisms have been suggested acting on both tumour initiation and progression. These potential mechanisms include modification of carcinogen activity, and enhancement of antioxidant and DNA repair processes [109].

### Occupation and occupational exposure to chemicals

Occupational factors are considered the second most important risk factor for bladder cancer after smoking [3, 110]. Over the years, many carcinogens have been identified by the IARC as definitely (group 1) or probably carcinogenic (group 2a). Occupational exposure to these carcinogens has been largely controlled and workers are now exposed to weaker carcinogens. The results of the MA by Cumberbatch et al. [53] show multiple occupations or occupational exposures that are significantly associated with the risk of bladder cancer.

Particularly workers exposed to aromatic amines, PAH, tobacco and tobacco smoke, combustion products, and heavy metals are at an increased risk. However, these findings will not be further discussed and repeated here as the MA by Cumberbatch et al. [61] offers an interesting and elaborate discussion on the matter. They also attempted to study a change in occupational risk over time but were limited by heterogeneity and small sample sizes.

Interestingly, farmers, gardeners, teachers, and forestry workers were found to be at a significantly lower risk. Farmers have been studied numerous times with regard to disease risk and have been suggested to be at both an increased and decreased risk of bladder cancer. It is thought of as a risk factor due to the exposure to chemicals such as pesticides, viruses, and other exposures. Although an increased risk was found in association with exposure to some pesticides, the evidence is not conclusive [111]. Both the European Food Safety Authority (EFSA) [112] and the IARC [113] did not find sufficient evidence to support carcinogenicity of pesticides. On the other hand, farmers, and possibly also gardeners and forestry workers, often have higher physical activity levels and fruit and vegetable consumption, as well as a lower prevalence of smoking [111].

Finally, in addition to exposure to carcinogens on an occupational basis, an increased risk of bladder cancer in certain occupational groups may also be the result of fewer possibilities for micturition during working hours. This may apply to, for example, sales workers, professional drivers, or other occupations where bathroom visits are limited to break times.

### Public health impact

The calculated POCs show that by adopting the right lifestyle, while considering harmful environmental and occupational exposures, a large proportion of the burden of bladder cancer could be prevented. Particularly for smoking the POC was high, highlighting that smoking cessation and prevention of smoking initiation should remain high on the agenda. In addition, a diet rich in fruit and vegetables, particularly citrus fruit and cruciferous vegetables, and low in processed meat should be stimulated. Particularly working conditions involving exposure to aromatic amines, PAHs, tobacco and tobacco smoke, combustion products, and heavy metals should be given priority to reduce exposure to these compounds.

### Limitations

The quality of a MA is strongly determined by the quality of the primary studies included. Not all studies corrected for smoking status, which is the most important risk factor for bladder cancer. The effects of this may be large, leading to an under- or overestimation of the true effect size. To minimise the effect of confounding in our study, only the most adjusted estimates from each MA were selected. Selection of the most comprehensive publication was based on the largest number of primary studies included, rather than the largest sample size. The latter would have been more desirable as the power of a study depends on it, but this data was often unavailable in the publications and number of studies included had to be used as a proxy. Results of MMA could be affected by duplicate inclusion of primary studies, leading to inflated precision and homogeneity [114]. Therefore, we minimised duplicate inclusion and performed a MMA only if at least 50 % of the included primary studies were ‘new’ in subsequent MAs.

The main limitation with regard to the combined POC is assumption of independence of risk factors. It is well known that lifestyle behaviours tend to cluster, [115] indicating that the factors are unlikely to be fully independent. Therefore, the combined POC is likely to be an overestimation and should be considered a maximum. However, the POCs of each of the individual factors are substantial, indicating that each plays an important part in the risk.

In this study, estimates of bladder cancer incidence were given priority over estimates of prevalence or mortality. Although the majority of included studies specified the outcome measure that was included or provided stratified results, not all did (Table 1). Because mortality and prevalence figures are greatly influenced by treatment success, this may have influence the estimates obtained.

Heterogeneity may always be an issue in MAs. Several of the studies included in the current review show moderate to high levels of heterogeneity. Whereas some identified study design, gender, or geographical region as possible source of heterogeneity [19, 22, 31], others could not find an explanation [20].

It should be noted that different classification criteria may be applied in studies to determine cases, including the TNM classification and stage grouping. Because any effect would be unlikely and small, this potential difference was not taken into consideration when selecting estimates.

This publication is a review of MAs. Additional risk factors for which no MA is available may prove to be of importance, but have not been included in this review. This study will help to identify gaps in knowledge with regard to MAs available.

It was our responsibility to give an objective and complete overview of the literature available. By using a protocol, including only MAs and by dealing appropriately with unexpected issues, we believe to have provided an overview of the risk factors associated with bladder cancer with high levels of objectivity and reliability.

In conclusion, the burden of bladder cancer could be significantly reduced through modification of lifestyle, environment, and occupational exposures. In fact, having numerous modifiable risk factors, among which multiple with a substantial RR, makes bladder cancer one of the most preventable diseases. Although substantial risks were found for, for example, smoking, some identified risk factors ‘only’ led to an increase or decrease of 10–15 %. This could be considered a minor effect, but reducing prevalence of a number of smaller risk factors together, could still result in significant overall risk reductions in the general population. Theoretically the included risk factors are, at least partially, modifiable. However, actually changing them is not always easily achieved, neither for one individual, let alone on a population level. Therefore, it is important that preventative and protective strategies pay attention to behaviour change in order to capture the greatest potential of risk reduction. When change is achieved, impact will not remain restricted to an improvement in the incidence of bladder cancer, as most of the included risk factors also play a role the risk of multiple other chronic diseases such as cancer or cardiovascular diseases, [116–118] and deaths [119]. Since relapse of bladder cancer is common, future research may also elucidate whether the same risk factors play a role in prevention of bladder cancer relapse as they do in incidence.

## Appendix 1

See Table 2.

**Table 2:** List of meta-analyses identified for modifiable risk factors of bladder cancer

Risk factor	Identified meta-analyses	Included	Included for?	Reason exclusion
*Diet*				
Fruit and vegetables	Riboli and Norat [120]	No		All primary studies are included in Yao et al. [19]
	Liu et al. [121]	No		>50 % overlap
	Vieira et al. [122]	No		>50 % overlap
	Liu et al. [123]	No		>50 % overlap
	Xu et al. [124]	No		>50 % overlap
	Steinmaus et al. [21]	Yes	Vegetables (MMA)	
	Yao et al. [19]	Yes	Fruit and vegetables Fruit Vegetables (MMA) Cruciferous vegetables	
	Liang et al. [20]	Yes	Citrus fruit	
Tea	Zeegers et al. [125]	No		Include other urinary tract cancer
	Zhang et al. [126]	No		>50 % overlap
	Wu et al. [127]	No		>50 % overlap
	Wang et al. [128]	No		>50 % overlap
	Boehm et al. [129]	No		Not MA
	Qin et al. [130]	Yes	Tea	
Coffee	Arab [131]	No		Not MA
	Pelucchi et al. [132]	No		Not MA
	Huang et al. [24]	No		>50 % overlap
	Sala et al. [28]	No		>50 % overlap
	Zhou et al. [25]	No		>50 % overlap
	Villanueva et al. [27]	Yes	Coffee (MMA)	
	Yu et al. [26]	Yes	Coffee (MMA)	
	Wu et al. [29]	Yes	Coffee (MMA)	
Beverage consumption	Zeegers et al. [6]	No		Not MA
	Boyle et al. [30]	Yes	Sweetened carbonated beverages	
Milk	Zhang et al. [133]	No		Not MA
	Lampe [134]	No		Not MA
	Mao et al. [31]	No		>50 % overlap
	Li et al. [135]	Yes	Milk	
Fish	Li et al. [32]	Yes	Fish	
Meat	Wang and Jiang [136]	No		Include urothelial cancer
	Steinmaus et al. [21]	No		
	Li et al. [33]	Yes	Meat	
Alcohol	Mao et al. [137]	No		Include urothelial cancer
	de Menezes et al. 138]	No		Not MA
	Bagnardi et al. [139]	No		>50 % overlap
	Zeegers et al. [140]	No		>50 % overlap
	Pelucchi et al. [34]	Yes	Alcohol	
Egg	Fang et al. [141]	No		Include urothelial cancer
	Li et al. [35]	Yes	Egg	
Dietary acrylamide	Pelucchi et al. [34]	Yes	Dietary acrylamide	
Vitamin A	Tang et al. [36]	Yes	Vitamin A	
	Steinmaus et al. [21]	No		
Vitamin D	Liao et al. [39]	Yes	Serum level	
	Chen et al. [40]	Yes	Diet and supplement	
Vitamin C & E	Wang et al. [38]	Yes	Vitamin C Vitamin E	
	Chen et al. [40]			>50 % overlap
Folate intake	He and Shui [41]	Yes	Folate	
Beta carotene supplement, Antioxidant supplement	Musa-Veloso et al. [142]	No		No MA for bladder cancer
	Coulter et al. [143]	No		No MA for bladder cancer
	Jeon et al. [37]	Yes	Beta-carotene supplements	
	Myung et al. [44]	Yes	Antioxidant supplement	
Selenium	Amaral et al. [42]	Yes	Selenium levels in the body	
	Vinceti et al. [43]	Yes	Selenium supplementation	
Omega-3 fatty acids	MacLean et al. [144]	No		No MA for bladder cancer
Zinc, Copper	Mao et al. [145]	No		No risk estimates reported
Olive oil	Psaltopoulou et al. [146]	No		No MA for bladder cancer
Fluid intake	Lotan et al. [147]	No		No MA
	Stelmach and Clasen [148]	No		No MA
	Villanueva et al. [27]	No		Included in Bai et al. [22]
	Isa (submitted)	No		>50 % overlap
	Bai et al. [22]	Yes	Total fluid intake	
Obesity	Qin et al. [47]	No		>50 % overlap
	Sun et al. [46]	Yes	Obesity	
*Behaviours*				
Smoking	Zeegers et al. [149]	No		Include other urinary tract cancer
	Crivelli et al. [150]	No		Effect of smoking on prognosis of bladder cancer patients
	Sasco et al. [151]	No		No MA
	Zeegers et al. [6]	No		No MA
	Vineis et al. [152]	No		No MA
	Shiels et al. [153]	No		Not primary prevention of BC
	‘t Mannetje et al. [154]	No		Discussed whether adjusting for smoking affects association between BC and occupation
	Freedman et al. [49]	No		>50 % overlap
	Brennan et al. [50]	No		>50 % overlap
	Brennan et al. (Women) [51]	No		>50 % overlap
	Hemelt et al. [11]	No		>50 % overlap
	Puente et al. [48]	No		>50 % overlap
	Cumberbatch et al. [53]	No		>50 % overlap
	Van Osch et al. [10]	Yes	Cigarette smoking	
Cigar and pipe	Pitard et al. [52]	Yes	Cigar and pipe	
Passive smoking	Van Hemelrijck et al. [54]	Yes	Passive smoking	
Smokeless tobacco	Lee et al. [55]	Yes	Smokeless tobacco	
Waterpipe tobacco smoking	Akl et al. [155]	No	Waterpipe tobacco smoking	No MA, reporting one CC study
Physical activity	Keimling et al. [56]	Yes	Physical activity	
*Environmental*				
Personal hair dyes use	Kelsh et al. [58]	No		All primary studies were included by Turati et al. [57]
	Takkouche et al. [60]	No		All primary studies were included by Turati et al. [57]
	Huncharek et al. [59]	No		All primary studies were included by Turati et al. [57]
	Rollison et al. [156]	No		No MA
	Turati et al. [57]	Yes	Personal hair dyes use	
*Occupational*				
Various occupations	Cumberbatch et al. [61]	Yes	Various occupations	
Various occupations	Reulen et al. [62]	No		Not the most comprehensive
Various occupations	Kogevinas et al. [63]	No		Not the most comprehensive
Various occupations	`t Mannetje et al. [64]	No		Not the most comprehensive
Painters	Bosetti et al. [157]	No		All primary studies are included by Bachand (2010)
	Kogevinas et al. [63]	No		All primary studies are included by Bosetti et al. [157]
	Guha et al. [158]	No		>50 % overlap
	Bachand et al. [159]	No		Not the most comprehensive
Hairdresser	Kogevinas et al. [63]	No		All primary studies included by Takkouche et al. [160]
	`t Mannetje et al. [64]	No		All primary studies included by Takkouche et al. [160]
	Takkouche et al. [160]	No		>50 % overlap
	Harling et al. [161]	No		Not the most comprehensive
Sales workers	Kogevinas et al. [63]	No		included in’t Mannetje et al. [160]
	Mannetje et al. [162]	No		>50 % overlap
Motor vehicle driving	Kogevinas et al. [63]	No		Included in Manju, 2009
	Boffetta and Silverman [163]	No		>50 % overlap
	Manju et al. [164]	No		Not the most comprehensive
Farmers	Acquavella et al. [165]	No		Not the most comprehensive
Foundry workers	Gaertner RR, 2002 [166]	No		Not the most comprehensive
Textile workers	Mastrangelo et al. [167]	No		Not the most comprehensive
Flight attendants	Tokumaru et al. [168]	No		>50 % overlap
	Buja et al. [169]	No		
Petroleum industry	Wong et al. [170]	No		Mortality estimate only
	Baena et al. [171]	No		Not the most comprehensive
Chemical workers	Greenberg et al. [172]	No		Not the most comprehensive
Dry cleaning	Vlaanderen et al. [173]	No		Not the most comprehensive
Inorganic lead compounds	Fu et al. [174]	No		Not the most comprehensive
Acrylonitrile workers	Cole et al. [175]	No		Included genitourinary cancers
	Collins and Acquavella [176]	No		Not the most comprehensive
Polycyclic aromatic hydrocarbons	Bosetti et al. [177]	No		Updated in Rota et al. [179]
	Boffetta et al. [178]	No		No MA
	Rota et al. [179]	No		Not the most comprehensive
Metalworking fluids	Calvert et al. [180]	No		No MA
Perchloroethylene (Dry cleaning)	Mundt et al. [181]	No		No MA
Asbestos-exposed workers	Goodman et al. [182]	No		Mortality estimate only

*CC* case–control, *MA* meta-analysis, *OR* odds ratio

## Appendix 2

See Table 3.

**Table 3:** Characteristics of meta-analyses investigating association between bladder cancer risk and various factors

References	Risk factor (s)	Primary studies included on bladder cancer in publication	Comparison	Selected as best estimate	Risk estimates	Number of primary studies included	Adjustment level	Heterogeneity	Publication bias (Egger test)
*Dietary factors*									
Yao et al. [19]	Fruit, vegetables	20 CC 11 COH	Fruit and vegetables; high versus low	Yes	RR 0.81; 95 % CI 0.67–0.99	10	Smoking	I^2^ = 58.4 %	
			Fruit; high versus low	Yes	RR 0.77; 95 % CI 0.69–0.87	27	Smoking	I^2^ = 54.9 %	Egger *p* = 0.003
			Vegetables; high versus low	Yes (MMA)	RR 0.81; 95 % CI 0.70–0.93	21	Smoking	I^2^ = 66.0 %	Egger *p* = 0.389
			Citrus Fruits; high versus low	No	RR 0.79; 95 % CI 0.68–0.91	11		I^2^ = 53.2 %	Egger *p* = 0.034
			Cruciferous vegetables; high versus low	Yes	RR 0.84; 95 % CI 0.77–0.91	11		I^2^ = 30.7 %	Egger *p* = 0.443
Steinmaus et al. [21]	Fruit (low intake)	38 CC and COH	Fruits; low versus high	No	RR 1.47; 95 % CI 1.25–1.74	8	Smoking	X^2^ *p* = 0.99^$^	
	Vegetables (low intake)		Vegetables; low versus high	Yes (MMA)	RR 1.15; 95 % CI 0.98–1.35	8	Smoking	X^2^ *p* = 0.82^$^	
	Meat		High versus low	No	RR 0.96; 95 % CI 0.73–1.27	4	Smoking	X^2^ *p* = 0.97^$^	
	Vitamin A		Retinol; low versus high intake	No	RR 1.01; 95 % CI 0.90–1.14	8		X^2^ *p* = 0.99^$^	
	Carotinoids		Carotenoids; low versus high intake	No	RR 1.10; 95 % CI 0.99–1.22	3		X^2^ *p* = 0.99^$^	
Vieira et al. [122]	Fruit, Vegetables	15 COH	Fruit and vegetables; high versus low	No	RR 0.89; 95 % CI 0.75–1.05	8		I^2^ = 33.8 %	
			Fruit; high versus low	No	RRs = 0.91; 95 % CI 0.82–1.00	11	Smoking	I^2^ = 11.0 %	Egger *p* = 0.48
			Vegetables; high versus low	No	RRs = 0.92; 95 % CI 0.84–1.01	9	Smoking	I^2^ = 5.0 %	Egger *p* = 0.02
			Citrus fruit; high versus low	No	RRs = 0.88; 95 % CI 0.77–1.01	6	Smoking	I^2^ = 0.0 %	Egger *p* = 0.38
			Cruciferous vegetables; high versus low	No	RRs = 0.85; 95 % CI 0.69–1.06	6	Smoking	I^2^ = 62.6 %	Egger *p* = 0.5
Liu et al. [123]	Fruit, vegetables	15 CC 12 COH	Fruit and vegetables; high versus low	No	RR 0.83; 95 % CI 0.69–1.01	9		I^2^ = 57.0 %	
			Fruit; high versus low	No	RR 0.81; 95 % CI 0.73–0.89	27		I^2^ = 53.7 %	Egger *p* = 0.052
			Vegetables; high versus low	No	RR 0.84; 95 % CI 0.72–0.96	24		I^2^ = 76.3 %	Egger *p* = 0.74
Xu et al. [124]	Fruit, vegetables	14 COH	Fruit and vegetables; every 0.2 serving increment per day	No	RR 1.00; 95 % CI 0.99–1.00	8		I^2^ = 38.5 %	
			Fruit; every 0.2 serving increment per day	No	RRs = 1.00; 95 % CI 0.99–1.00	14	Smoking	I^2^ = 45.8 %	Egger *p* < 0.01
			Vegetables; every 0.2 serving increment per day	No	RRs = 1.00; 95 % CI 0.99–1.00	14	Smoking	I^2^ = 19.0 %	Egger *p* = 0.93
			Citrus fruit; every 0.2 serving increment per day	No	RRs = 1.00; 95 % CI 1.00–1.00	7		I^2^ = 0.0 %	
			Cruciferous vegetables; every 0.2 serving increment per day	No	RRs = 0.97; 95 % CI 0.93–1.01	8		I^2^ = 55.8 %	
Liang et al. [20]	Citrus fruits	8 CC 6 COH	Citrus fruits; high versus low	Yes	RR 0.85; 95 % CI 0.76–0.94	14		I^2^ = 72.1 %	Egger *p* = 0.004
		6 COH	Citrus fruits; high versus low	No	RR 0.96; 95 % CI 0.87–1.07	6		I^2^ = 19.8 %	
		8 CC	Citrus fruits; high versus low	No	RR 0.77; 95 % CI 0.64–0.92	8		I^2^ = 68.7 %	
Liu et al. [121]	Cruciferous vegetables	5 CC 5 COH	Cruciferous vegetables; high versus low	No	RR 0.80; 95 % CI 0.69–0.92	10		I^2^ = 45.9 %	Egger *p* = 0.51
		5 COH	Cruciferous vegetables; high versus low	No	Cohort: RR 0.86; 95 % CI 0.61–1.11	5		I^2^ = 73 %	
		5 CC	Cruciferous vegetables; high versus low	No	CC: RR 0.78; 95 % CI 0.67–0.89	5		I^2^ = 0.0 %	
Bai et al. [22]	Total fluid intake	17 CC 4 COH	High versus low	Yes	RRm = 1.12; 95 % CI 0.94–1.33	14	Most adjusted	I^2^ = 82.8 %	Egger *p* = 0.059
Isa (submitted)	Total fluid intake	15 CC 3 COH	250 ml/day increment	No	RR 1.02; 95 % CI 1.00–1.04	15			Egger *p* = 0.614
Zhang et al. [126]	Tea	7 COH	All tea; high versus low	No	RR 0.92; 95 % CI 0.76–1.13	7		I^2^ = 23.8 %	
			All tea; one cup/day increment	No	RR 0.98; 95 % CI 0.93–1.02	7		I^2^ = 44.1 %	
			Green tea; one cup/day increment	No	RR 1.02; 95 % CI 0.95–1.11	3		I^2^ = 63.9 %	
			Black tea; one cup/day increment	No	RR 0.73; 95 % CI 0.33–1.60	1			
Wu et al. [127]	Tea	12 CC 3 COH	All tea; high versus low	No	RRm = 1.01; 95 % CI 0.80–1.29	7	Most adjusted		Egger *p* = 0.88
			Green tea; high versus low	No	RR 1.03; 95 % CI 0.82–1.31	5		I^2^ = 0.0 %	Egger *p* = 0.49
			Black tea; high versus low	No	RR 0.84; 95 % CI 0.7–1.01	6		I^2^ = 0.0 %	Egger *p* = 1.0
Wang et al. [128]	Tea	13 CC 4 COH	All tea; high versus low	No	RRm = 1.12; 95 % CI 0.88–1.43; I^2^ = 64.6 %; n = 17	17	Most adjusted		Egger *p* = 0.298
			Green tea; high versus low	No	RR 0.81; 95 % CI 0.68–0.98	4		I^2^ = 0.0 %	Egger *p* = 0.73
			Black tea; high versus low	No	RR 1.05; 95 % CI 0.83–1.32	5		I^2^ = 49.4 %	Egger *p* = 0.41
Qin et al. [130]	Tea	17 CC 6 COH	All tea; high versus low	Yes	RR 0.94; 95 % CI 0.85–1.04	23		I^2^ = 28.3 %	Egger *p* = 0.52
			Green tea; high versus low	No	RR 0.97; 95 % CI 0.73–1.21;	5		I^2^ = 0.0 %	Egger *p* = 0.38
			Black tea; high versus low	No	RR 0.79; 95 % CI 0.59–0.99	7		I^2^ = 33.1 %	Egger *p* = 0.38
Huang et al. [24]	Coffee	5 COH	High versus none/low	No	RR 1.08; 95 % CI 0.71–1.66	5		I^2^ = 62.9 %	
Zhou et al. [25]	Coffee	20 CC 5 COH	High (>5–6 cups/day) versus none	No	Case–control: RRs = 1.45; 95 % CI 1.29–1.63 This category was not calculated for cohort studies	20		I^2^ = 31.8 %	No publication bias (no test estimate reported)
Sala et al. [28]	Coffee	Pooled analysis of 10 CC	6–9 cups versus none	No	RRm = 1.0; 95 % CI 0.6–1.4		Most adjusted, analysis restricted to non-smokers		
			≥10 cups versus none	No	RRm = 1.8; 95 % CI 1.0–3.3		Most adjusted, analysis restricted to non-smokers		
Villanueva et al. [27]	Coffee	Pooled analysis of 6 CC	>5 cups/day versus ≤5 cups/day; overall	Yes (MMA)	RRms = 1.25; 95 % CI 1.08–1.44	6	Most adjusted, incl. smoking		
			>5 cups/day versus ≤5 cups/day; men only	No	RRms = 1.23; 95 % CI 1.05–1.44	5	Most adjusted, incl. smoking		
			>5 cups/day versus ≤5 cups/day; women only	No	RRms = 1.31; 95 % CI 0.99–1.74	5	Most adjusted, incl. smoking		
			1 l/day increment	No	RRms = 1.24; 95 % CI 1.08–1.43	6	Most adjusted, incl. smoking		
Yu et al. [26]	Coffee	9 COH	Drinkers versus none/lowest	Yes (MMA)	RR 0.83; 95 % CI 0.73–0.94	9		I^2^ = 39.3 %	Egger *p* = 0.793
Wu et al. [29]	Coffee	34 CC 6 COH	High versus low	Yes (MMA)	RRms = 1.28; 95 % CI 1.21–1.46	18	Most adjusted, incl. smoking	I^2^ = 28.5	Egger *p* = 0.051
Boyle et al. [30]	Sweetened carbonated beverages	5 studies	High versus low	Yes	RR 1.13; 95 % CI 0.89–1.45	5		I^2^ = 0.0 %	Egger *p* = 0.05
Li et al. [135]	Milk Dairy products	12 CC 6 COH	Milk; high versus low	Yes	RRm = 0.91; 95 % CI 0.72–1.15	7	Most adjusted	Q *p* = 0.020	Egger *p* = 0.048
			Dairy products; high versus low	Yes	RRm = 1.01; 95 % CI 0.86–1.19	3	Most adjusted	Q *p* = 0.108	Egger *p* = 0.73
Mao et al. [31]	Milk and Dairy products	8 HCC 5 PCC 6 COH	All milk; high versus low	No	RRms = 0.84; 95 % CI 0.72–0.97	16	Most adjusted, incl. smoking	I^2^ = 70.1 %	Egger *p* = 0.055
			Whole milk; high versus low	No	RR 2.23; 95 % CI 1.45–3.00	2		I^2^ = 0	
			Skim milk; high versus low	No	RR 0.47; 95 % CI 0.18–0.79	2		I^2^ = 0	
			Fermented milk; high versus low	No	RR 0.69; 95 % CI 0.47–0.91	5		I^2^ = 62.5 %	
Li et al. [32]	Fish	6 HCC 3 PCC 5 COH	High versus low	Yes	RRs = 0.86; 95 % CI 0.61–1.12; I^2^ = 85.4 %; n = 14; Begg’s *p* = 0.101	14	Smoking		
Li et al. [33]	Meat	9 CC 5 COH	Red meat; high versus low	Yes	RR 1.15; 95 % CI 0.97–1.36	14		I^2^ = 73.5 %	Egger *p* = 0.83
		6 CC 5 COH	Processed meat; high versus low	Yes	RR 1.22; 95 % CI 1.04–1.43	11		I^2^ = 64.9 %	Egger *p* = 0.35
Pelucchi et al. [34]	Alcohol	14 CC 2 NCC 3 COH	Heavy intake, ≥ 3 drinks (≥ 37.5 g)/day versus non-drinkers	Yes	RR 0.97; 95 % CI 0.72–1.31	7	Smoking	X^2^ *p* < 0.01	
			Moderate intake, <3 drinks/day versus non-drinkers	No	RR 0.98; 95 % CI 0.89–1.07	15	Smoking	X^2^ *p* = 0.05	
Bagnardi et al. [139]	Alcohol	9 CC 2 COH	Highest category (100 g/day) versus non-drinkers	No	RR 1.17; 95 % CI: 0.97–1.41	11		Het. *p* = N.S.	
			Highest category (100 g/day) versus non-drinkers	No	RRs = 1.09; 95 % CI: 0.89–1.33		Smoking	Het. *p* < 0.05	
Zeegers et al. [140]	Alcohol	6 studies	Current alcohol use versus non-use	No	RR 1.3; 95 % CI 1.1–1.5	6			
Li et al. [35]	Egg	9 CC 4 COH	All eggs; high versus low	Yes	RRm = 1.11; 95 % CI 0.73–1.69	6	Most adjusted	I^2^ = 75.8 %	Egger *p* = 0.55
			Fried eggs; high versus low	No	RR 2.04; 95 % CI 1.41–2.95	2		I^2^ = 0.0 %	
			Boiled eggs; high versus low	No	RR 1.25; 95 % CI 0.82–1.91	2		I^2^ = 35.5 %	
Pelucchi et al. [45]	Dietary acrylamide	1 CC 1 COH 1 C-COH	High versus low	Yes	RR 0.93; 95 % CI 0.78–1.11	3		X^2^ *p* = 0.91	
			10 mg/day increase in intake	No	RR 1.00; 95 % CI 0.96–1.03	3		X^2^ *p* = 0.97	
Tang et al. [36]	Vitamin A (total)	14 CC 11 COH	Supplement, diet and blood levels; high versus low	Yes	RRms = 0.82; 95 % CI 0.65–0.95	11	Most adjusted, incl. smoking	I^2^ = 46.3 %	Egger *p* = 0.057
			Dietary intake; high versus low	No	RRms = 0.90; 95 % CI 0.80–1.01		Most adjusted, incl. smoking	I^2^ = 0 %	
			Supplementation versus placebo or no supplementation	Yes	RRms = 0.64; 95 % CI 0.47–0.82		Most adjusted, incl. smoking	I^2^ = 0 %	
Jeon et al. [37]	Beta-carotene supplements	2 RCT	Supplementation versus placebo or no supplementation	No	RR 1.52; 95 % CI 1.03–2.24	2		I^2^ = 0.0 %	
Liao et al. [39]	Vitamin D	1 CC 2 NCC 2 COH	Serum levels; high versus low	Yes	RRs = 0.75; 95 % CI 0.65–0.87	5	Smoking	I^2^ = 0.0 %	Egger *p* = 0.57
Chen et al. [40]	Vitamin D	1 CC 3 COH	Circulating levels; high versus low	No	RR 0.75; 95 % CI 0.57–0.99	4		I^2^ = 51.7 %	Egger *p* = 0.87
		3 studies	Diet and supplement; high versus low	No	RR 0.92; 95 % CI 0.66–1.28	3		I^2^ = 32.3 %	Egger *p* = 0.41
	Vitamin E	4 CC 5 COH	Diet; high versus low	No	RRm = 0.69; 95 % CI 0.52–0.92	5		I^2^ = 47.1 %	Egger *p* = 0.01
		3 CC 3 COH	Diet and supplement; high versus low	No	RRm = 0.76; 95 % CI 0.56–1.02	5		I^2^ = 49.8 %	Egger *p* = 0.35
		1 CC 6 COH	Supplementation versus placebo or no supplementation	No	RRm = 0.64; 95 % CI 0.48–0.85			I^2^ = 0.6 %	Egger *p* = 0.39
	Vitamin C	7 CC 7 COH	Diet; High versus Low	No	RRm = 0.69; 95 % CI 0.58–0.82	6		I^2^ = 13.7 %	Egger *p* = 0.28
		5 CC 3 COH	Diet and supplement; high versus low	No	RRm = 0.80; 95 % CI 0.62–1.03	5		I^2^ = 33.4 %	Egger *p* = 0.03
		6 CC 3 COH	Supplementation versus placebo or no supplementation	No	RRm = 0.84; 95 % CI 0.55–1.29	4		I^2^ = 74.4 %	Egger *p* = 0.002
Wang et al. [38]	Vitamin C	11 CC 9 COH	Intake; high versus low	Yes	RRs = 0.90; 95 % CI 0.79–1.00	20	Smoking	I^2^ = 43.7 %	Egger *p* = 0.064
	Vitamin E	7 CC 8 COH	Intake; high versus low	Yes	RRs = 0.82; 95 % CI 0.74–0.90	15	Smoking	I^2^ = 0 %	Egger *p* = 0.534
He et al. [41]	Folate	6 CC 7 COH	Intake; high versus low	Yes	RR 0.84; 95 % CI 0.72–0.96	13		I^2^ = 28.9 %	Egger *p* = 0.06
Myung et al. [37]	Antioxidant supplement	4 RCT	Supplementation versus placebo or no supplementation	No	RR 1.52; 95 % CI 1.06–2.17	4		I^2^ = 0.0 %	
Amaral et al. [42]	Selenium	3 CC 3 NCC 1 C-COH	Serum and toenail levels; high versus low	Yes	OR = 0.61; 95 % CI 0.42–0.87	7		I^2^ = 60.8 %	Egger *p* = 0.357
Vinceti et al. [43] (Cochrane SR)	Selenium	5 prospective studies	Serum, toenail or plasma levels; high versus low	No	OR = 0.67; 95 % CI 0.46–0.97	5		I^2^ = 30 %	
		2 RCT	Supplementation versus Placebo	Yes	RR 1.14; 95 % CI 0.81–1.61	2		I^2^ = 0.0 %	
Sun et al. [46]	Obesity	15 COH	Obese versus normal weight	Yes	RRs = 1.10; 95 % CI 1.03–1.18	12	Smoking	I^2^ = 8.8 %	Egger *p* = 0.712
			Pre-obese versus normal weight	Yes	RRs = 1.07; 95 % CI 0.99–1.16	13	Smoking	I^2^ = 46.1 %	Egger *p* = 0.712
Qin et al. [47]	Obesity	11 COH	Obese versus normal weight	No	RRs = 1.09; 95 % CI 1.01–1.17	9	Smoking	I^2^ = 35.9 %	Egger *p* = 0.244
*Behavioural factors*									
Hemelt et al. [11]	Smoking	21 CC 11 COH 2 NCC	All tobacco; Ever smokers versus never smokers	No	RR 2.25; 95 % CI 1.96–2.59	15			
			All tobacco; Ex smokers versus never smokers	No	RR 1.90; 95 % CI 1.71–2.11	8			
			All tobacco; Current smokers versus never smokers	No	RR 3.35; 95 % CI 2.90–3.88	11			
			Cigarettes; Ever smokers versus never smokers	No	RR 2.24; 95 % CI 1.81–2.78	9			
			Cigarettes; Ex smokers versus never smokers	No	RR 1.95; 95 % CI 1.55–2.47	4			
			Cigarettes; Current smokers versus never smokers	No	RR 3.13; 95 % CI 2.33–4.21	6			
Puente et al. [48]	Smoking	Pooled analysis of 14 CC	Current smokers versus never smokers; Male	No	RR 3.89; 95 % CI 3.53–4.29				
			Current smokers versus never smokers; Female	No	RR 3.55; 95 % CI 3.06–4.10				
			Ex-smokers versus never smokers; Male	No	RR 2.21; 95 % CI 2.01–2.43				
			Ex-smokers versus never smokers; Female	No	RR 2.21; 95 % CI 1.87–2.61				
Freedman et al. [49]	Smoking	7 COH	Current smokers versus never smokers	No	RR 2.94; 95 % CI 2.45–3.54	7		I^2^ = 0.0 %	Egger *p* = 0.32
Brennan et al. [50]	Smoking	Pooled analysis of 11 CC (men only)	Ever-smokers versus never smokers	No	RR 3.63; 95 % CI 3.13–4.20				
			Current smokers versus never and ex-smokers	No	RR 2.47; 95 % CI 2.23–2.74				
Brennan et al. [51]	Smoking	Pooled analysis of 11 CC (women only)	Ever-smokers versus never smokers	No	RR 3.1; 95 % CI 2.5–3.9				
			Current smokers versus never and ex-smokers	No	RR 2.9; 95 % CI 2.3–3.7				
Van Osch et al. submitted [10]	Active smoking	18 COH 71 CC	Active smoking; Ex-smokers versus never smokers	Yes	RR 1.83; 95 % CI 1.52–2.14	12	Age, sex		Egger *p* = 0.150
			Active smoking; Current smokers versus never smokers	Yes	RR 3.14; 95 % CI 2.53–3.75	13	Age, sex		
			Active smoking; ≤20 versus >20 years at first exposure	No	RR 1.33; 95 % CI 0.92–1.74	4			
Cumberbatch et al. [53]	Active smoking		Ex-smokers versus never smokers	No	RR 3.37; 95 % CI 3.01–3.78	48	Most adjusted estimates in MA	I^2^ = 82.2 %	Egger *p* = 0.13 Begg *p* = 0.03
			Current smokers versus never smokers	No	RR 1.98; 95 % CI 1.76–2.22	49	Most adjusted estimates in MA	I^2^ = 78.6 %	
			Pipe; Smokers versus never smokers	No	RR 1.49; 95 % CI 1.18–1.88	4		I^2^ = 39.4 %	
			Cigar; Smokers versus never smokers	No	RR 1.62; 95 % CI 1.18–2.22	4		I^2^ = 0.0 %	
	Smokeless tobacco		Snuff; users versus non-users	No	RR 0.89; 95 % CI 0.56–1.42	2		I^2^ = 23.7 %	
			Chewing tobacco; users versus non-users	No	RR 1.04; 95 % CI 0.75–1.45	2		I^2^ = 0.0 %	
Pitard et al. [52]	Active smoking	Pooled analysis of 6 CC (men only)	Pipe only; Smokers versus never smokers	Yes	RR 1.9; 95 % CI 1.2–3.1				
			Cigar only; Smokers versus never smokers	Yes	RR 2.3; 95 % CI 1.6–3.5				
			Cigarette only; Smokers versus never smokers	No	RR 3.5; 95 % CI 2.9–4.2				
Van Hemelrijck et al. [54]	Passive smoking	5 CC 3 COH	Exposed versus not exposed	Yes	RR 0.99; 95 % CI 0.86–1.14	8	Non-smokers only	I^2^ = 35.6 %	Begg *p* = 0.32
Lee et al. [55]	Smokeless tobacco	12 CC 1 COH	Smokeless tobacco use versus never use	Yes	RRs = 0.95; 95 % CI 0.71–1.29	9	Smoking	I^2^ = 59.6 %	
Keimling et al. [56]	Physical activity	4 CC 11 COH	High versus low	Yes	RRm = 0.86; 95 % CI 0.77–0.95	7	Most adjusted	I^2^ = 0.0 %	
Turati et al. [57]	Personal hair dye	15 CC 2 COH	Personal use of any type of hair dyes versus no use	Yes	RRs = 0.94; 95 % CI 0.82–1.08	12	Smoking	I^2^ = 49.9 %	Egger *p* = 0.54
Occupational (Comparing the specific occupation versus any other occupation or the general population)									
Guha et al. [158]	Painters	30 CC 2 COH 9 RL	Painters	No	RR 1.25; 95 % CI 1.16–1.34	41		I^2^ = 23.5 %	
			Painters	No	RRs = 1.28; 95 % CI 1.15–1.43		Smoking	I^2^ = 0.7 %	
			Painters	No	RRms = 1.27; 95 % CI 0.99–1.63		Most adjusted, incl. smoking	I^2^ = 0.1 %	
Bachand et al. [159]	Painters	33 CC	Painters	No	RR 1.30; 95 % CI 1.17–1.44	33	Smoking; morbidity and mortality combined		
		4 COH	Painters	No	RR 1.23; 95 % CI 0.95–1.59	4	Smoking; morbidity only		
Harling et al. [161]	Hairdressers	28 CC 14 RCOH	Hairdressers	No	RR 1.35; 95 % CI 1.13–1.61	23	Smoking	X^2^ *p* = 0.19	
			Hairdressers; job held ≥ 10 years	No	RR 1.70; 95 % CI 1.01–2.88	6		X^2^ *p* = 0.46	
Takkouche et al. [160]	Hairdressers	26 CC 8 COH	Hairdressers	No	RR 1.33; 95 % CI 1.07–1.67	19	Smoking	Q *p* = 0.77	
			Hairdressers	No	RR 1.35; 95 % CI 1.21–1.50	26	Incidence only	Q *p* = 0.78	
‘t Mannetje et al. [162]	Sales workers	10 CC 2 RL 3 mortality studies	Sales workers; Men	No	RR 1.04; 95 % CI 0.97–1.12		Smoking, incidence only	Q *p* = 0.3	Egger *p* = 0.4
			Sales workers; Women	No	RR 1.22; 95 % CI 1.06–1.41		Smoking, incidence only	Q *p* = 0.44	Egger *p* < 0.01
Manju L et al. [164]	Motor vehicle driving	3 COH	Overall (motor vehicle drivers and railroad workers)	No	RR 1.08; 95 % CI 1.00–1.17	3		Het. *p* = 0.85	
		27 CC	Truck drivers	No	RR 1.18; 95 % CI 1.09–1.28	18		Het. *p* = 0.25	
		27 CC	Bus drivers	No	RR 1.23; 95 % CI 1.06–1.44	11		Het. *p* = 0.19	
Boffetta and Silverman [163]	Motor vehicle driving	16 CC 7 COH 6 routinely collected data (men only)	Truck drivers	No	RR 1.17; 95 % CI 1.06–1.29	15		Het. *p* = 0.3	Egger *p* = 0.07
			Bus drivers	No	RR 1.33; 95 % CI 1.22–1.45	10		Het. *p* = 0.4	Egger *p* = 0.001
Acquavella et al. [165]	Farmers	10 Follow-up studies 8 CC 11 PMR (men only)	Farmers	No	RR 0.79; 95 % CI 0.72–0.86	29		X^2^ *p* < 1*10^−5^	
		10 Follow-up studies	Farmers	No	RR 0.62; 95 % CI 0.59–0.65	10		X^2^ *p* = 0.08	
		8 CC	Farmers	No	RR 0.94; 95 % CI 0.86–1.04	8		X^2^ *p* = 0.09	
Gaertner et al. [166]	Foundry workers	22 CC 9 COH 14 surveillance studies	Foundry workers	No	RR 1.11; 95 % CI 0.96–1.29	16 (CC only)	Smoking	X^2^ *p* = 0.07 (15df)	
			Foundry workers	No	RR 1.14; 95 % CI 1.04–1.26;	11	Incidence only	X^2^ *p* = 0.28 (10df)	
Mastrangelo et al. [167]	Textile workers	Unknown	Spinners	No	RR 1.19; 95 % CI 0.80–1.57	2		X^2^ *p* < 0.001^$^	
			Weavers	No	RR 2.40; 95 % CI 1.62–3.18	2		X^2^ *p* = 0.001^$^	
			Dyers	No	RR 1.39; 95 % CI 1.07–1.71	3		X^2^ *p* = 0.505^$^	
Tokumaru et al. [168]	Flight attendants	3 COH (female only)	Flight attendants	No	RR 2.03; 95 % CI 0.75–5.43	3	Incidence only	Q *p* = N.S.	
Buja et al. [169]	Flight attendants	4 COH (female only)	Flight attendants	No	RR 1.45; 95 % CI 0.33–3.16	4	Incidence only	Tau = 0.39	
Baena et al. [171]	Petroleum industry	8 CC	Petroleum industry workers	No	RR 1.5; 95 % CI 1.29–1.75	8			
Greenberg et al. [172]	Chemical workers	19 COH for incidence	Chemical workers	No	RR 2.21; 95 % CI 1.18–4.15	19	Incidence only		
Vlaanderen et al. [173]	Dry cleaning	4 CC 3 COH	Dry cleaning workers	No	RR 1.47; 95 % CI 1.16–1.85	7		I^2^ = 0 %	Egger *p* > 0.05
Fu et al. [174]	Inorganic lead compounds	5 studies	Exposed to inorganic lead compounds versus non-exposed	No	RR 1.41; 95 % CI 1.16–1.71	5		Hom. *p* > 0.30	
Collins et al. [176]	Acrylonitrile workers	10 studies	Acrylonitrile workers	No	RR 0.8; 95 % CI 0.3–2.2	3	Incidence	Het. *p* = 0.49	
Rota et al. [179]	Polycyclic aromatic hydrocarbons (PAHs)	13 COH	Aluminium production workers	No	RR 1.28; 95 % CI 0.98–1.68	10		X^2^ *p* = 0.002	Egger *p* = 0.22
			Asphalt workers	No	RR 1.03; 95 % CI 0.82–1.30	2		X^2^ *p* = 0.31	
			Carbon black production	No	RR 1.10; 95 % CI 0.61–2.00	3		X^2^ *p* = 0.29	Egger *p* = 0.61
			Workers in iron and steel foundry	No	RR 1.38; 95 % CI 1.00–1.91	9		X^2^ *p* = 0.001	Egger *p* = 0.3

*95* *% CI* 95 % confidence interval, *CC* case–control study, *C-COH* case-cohort study, *COH* cohort study, *df* degrees of freedom, *ECO* ecological study, *HCC* hospital-based case–Control study, *Het.* heterogeneity, *Hom.* homogeneity, *NCC* nested case–control study, *N.S.* not significant, *OR* odds ratio, *PCC* population-based case–control study, *PCOH* prospective cohort study, *PMR* proportional mortality ratio, *RCOH* retrospective cohort study, *RE* risk estimate, *RCT* randomised controlled trial, *RL* record linkage study, *RR* relative risk, *SIR* standardised incidence ratio

^$^The *p* value was calculated by the authors of the current article when only the *X* ^2^ statistic and degrees of freedom were presented

## Appendix 3

See Table 4.

**Table 4:** Risk estimates for occupations reported by Cumberbatch et al., Reulen et al., Kogevinas et al., and ‘t Mannetje et al

Cumberbatch et al. [53] (Meta-analysis of 217 studies)		Reulen et al. [62] (Meta-analysis of 66 cohort studies and 64 case–control studies)		Kogevinas et al. [63] (Pooled analysis of 11 European CC studies)		‘t Mannetje et al. [64] (Pooled analysis of 11 European CC studies)	
Occupation	RRs (95 % CI)	Occupation	RRs (95 % CI)	Occupations (ISCO code*)[183]	RR (95 % CI)	Occupations (ISCO codes)	RR (95 % CI)
*Occupations with statistically significant results*							
Tobacco workers	1.72 (1.37–2.15)	Blacksmiths	1.58 (1.05–2.36)	Working proprietors—wholesale and retail trade (41)	1.33 (1.04–1.69)	Blacksmiths, toolmakers and machine-tool operators (83)	1.9 (1.1–3.6)
Dye workers	1.58 (1.32–1.90)	Blacksmiths and tool-makers	1.22 (1.09–1.35)	Working proprietor—retail trade (41030)	1.35 (1.02–1.79)	Lathe operator (83420)	4.6 (1.1–19.2)
Chimney sweeps	1.53 (1.30–1.81)	Building finishers	1.16 (1.02–1.33)	Working proprietor—cafe. bar and snack bar (51050)	2.2 (1.16–4.20)	Blacksmiths, toolmakers and machine-tool operators nec. (839)	2.9 (1.3–6.3)
Nurses	1.49 (1.06–2.08)	Domestic helpers	1.39 (1.14–1.70)	Concierge—apartment house (55120)	2.64 (1.10–6.32)	Field crop and vegetable farm workers (622)	1.8 (1.0–3.1)
Rubber workers	1.49 (1.37–1.61)	Dye makers	1.14 (1.09–1.19)	Janitor (55130)	2.28 (1.30–3.98)	Tobacco prepares and tobacco product makers (78)	3.1 (1.1–9.3)
Waiters	1.43 (1.34–1.52)	Furnace operators	1.41 (1.11–1.79)	Nursery workers and gardeners (627)	1.5 (1.07–2.10)	Tailors and dressmakers (791)	1.4 (1.0–2.1)
Aluminum workers	1.41 (1.29–1.55)	Glass-makers	1.69 (1.13–2.52)	Other nursery workers and gardeners (62790)	3.57 (1.24–10.29)	Other saleswomen, shop assistants and demonstrators (45190)	2.6 (1.0–6.9)
Hairdressers	1.32 (1.24–1.40)	Launderers	1.72 (1.25–2.37)	Supervisor and general foreman—metal processing—(70030)	2.11 (1.04–4.32)	Mail sorting clerk (37020)	4.4 (1.0–19.5)
Printers	1.23 (1.17–1.30)	Leather workers	1.37 (1.10–1.70)	Supervisor and general foreman—manufacturing of machinery and metal products (70050)	1.59 (1.05–2.42)		
Seamen	1.23 (1.17–1.29)	Machine-tool setters	1.31 (1.12–1.53)	Miners, quarrymen, well drillers and related workers (71)	1.26 (1.00–1.58)		
Oil and petroleum workers	1.2 (1.06–1.37)	Machinist	1.16 (1.04–1.29)	Miners and quarrymen (711)	1.3 (1.02–1.64)		
Shoe and leather workers	1.2 (1.12–1.29)	Mechanics	1.20 (1.06–1.35)	Metal casters (724)	1.96 (1.06–3.64)		
Plumbers	1.2 (1.14–1.27)	Metal processors	1.24 (1.11–1.38)	Metal processers NEC (729)	1.85 (1.15–2.97)		
Sales agents	1.17 (1.15–1.20)	Metal workers	1.15 (1.01–1.32)	Knitters (755)	2.56 (1.24–5.30)		
Artistic workers	1.16 (1.10–1.22)	Miners	1.57 (1.21–2.03)	Knitting-machine operator-hosiery (75530)	3.26 (1.26–8.40)		
Cooks and stewards	1.15 (1.08–1.22)	Motor mechanics	1.39 (1.11–1.73)	Metal workers and machinists (832–835, 841, 849)	1.16 (1.02–1.32)		
Chemical process workers	1.14 (1.10–1.19)	Paper-pulp workers	1.29 (1.02–1.63)	Machine-tool setter-operators (833)	1.5 (1.07–2.12)		
Metal workers	1.14 (1.11–1.18)	Rubber workers	1.43 (1.18–1.71)	Metalworking machine setter—general (83305)	2.27 (1.03–5.00)		
Drivers	1.14 (1.11–1.16)	Writers and artists	1.34 (1.06–1.71)	Metalworking machine setter-operator—general—(83310)	3.35 (1.19–9.44)		
Fishermen	1.13 (1.08–1.19)			Precision-grinding-machine setter-operator—(83370)	5.21 (1.48–18.31)		
Painters	1.13 (1.09–1.17)			Machinery fitters, machine assemblers and precision-instrument makers—except electrical (84)	1.16 (1.01–1.34)		
Assistant nurses	1.12 (1.04–1.20)			Automobile mechanic (84320)	1.38 (1.02–1.87)		
Domestic assistants	1.12 (1.07–1.18)			Machinery fitters, machine assemblers and precision-instrument makers—except electrical—NEC (849)	1.27 (1.03–1.55)		
Launderers and dry cleaners	1.12 (1.04–1.21)			Textile machinery mechanic (84945)	2.86 (1.50–5.47)		
Public safety workers–police	1.11 (1.07–1.16)			Other electrical fitters (85190)	3.99 (1.10–14.51)		
Physicians	1.11 (1.03–1.19)			Electric arc welder—hand (87220)	2.27 (1.04–4.98)		
Clerical workers	1.11 (1.10–1.13)			Printers and related workers (92)	1.45 (1.07–1.97)		
Electrical workers	1.11 (1.07–1.14)			Printing pressmen (922)	1.81 (1.03–3.17)		
Military personnel	1.11 (1.05–1.18)			Automobile painter (93960)	1.95 (1.01–3.75)		
Mechanics	1.11 (1.09–1.13)			Excavating-machine operator (97420)	2.43 (1.18–5.00)		
Smelting workers	1.11 (1.06–1.16)			Transport equipment operators (98)	1.17 (1.02–1.34)		
Transport workers	1.1 (1.06–1.13)						
Glass makers, etc.	1.1 (1.05–1.15)						
Textile workers	1.1 (1.06–1.14)						
Waiters and bartenders	1.1 (1.01–1.19)						
Building caretakers	1.09 (1.06–1.13)						
Health care workers	1.09 (1.06–1.12)						
Food manufacturing workers	1.08 (1.04–1.12)						
Postal workers	1.08 (1.03–1.13)						
Packers, loaders, and warehouse workers	1.08 (1.04–1.13)						
Shop workers	1.07 (1.05–1.10)						
Technical workers, etc.	1.04 (1.02–1.06)						
Economically inactive	0.96 (0.95–0.97)						
Religious and legal workers, etc.	0.93 (0.88–0.97)						
Forestry workers	0.88 (0.86–0.90)						
Teachers	0.85 (0.82–0.87)						
Gardeners	0.78 (0.75–0.81)						
Farmers	0.69 (0.68–0.71)						
*Occupations with no statistically significant results*							
Beverage workers	1.35 (0.84–2.16)	Architects and engineers	0.99 (0.85–1.16)	Medical doctors (061)	0.82 (0.40–1.69)	Hairdressers, barbers, beauticians and related workers (57)	0.8 (0.4–1.7)
Iron and metal ware workers	1.18 (0.98–1.42)	Armed forces	1.09 (0.90–1.33)	Nurses (071–079)	0.89 (0.51–1.56)	Chemical processors and related workers (74)	0.6 (0.2–2.2)
Other health workers	1.11 (0.97–1.27)	Bakers	1.02 (0.54–1.92)	Teachers (13)	1 (0.74–1.34)	Spinners, weavers, knitters, dyers and related workers (75)	0.9 (0.6–1.3)
Dentists	1.09 (0.96–1.23)	Bricklayers	1.15 (0.96–1.38)	Salesmen, shop assistants (45)	0.97 (0.82–1.14)	Shoe makers and leather goods makers (80)	0.4 (0.2–1.1)
Welders	1.06 (1.00–1.12)	Building caretakers	1.26 (0.88–1.80)	Cooks, waiters, bartenders (53)	1..19 (0.91–1.55)	Metal working including toolmakers, machine-tool setter-operators, metal grinders and machinery fitters (832–835, 841, 849)	1.5 (0.8–2.7)
Bricklayers	1.05 (0.99–1.12)	Building frame workers	1.08 (0.93–1.26)	Launderers, dry-cleaners and pressers (56)	1.24 (0.67–2.31)	Rubber and plastics product makers (90)	1.2 (0.5–3.0)
Miners and quarry workers	1.05 (1.00–1.11)	Butchers	1.38 (0.88–2.18)	Hairdressers (57)	1.09 (0.70–1.70)		
Laboratory assistants	1.04 (0.92–1.18)	Cabinet makers	0.98 (0.58–1.67)	Fire-fighters (581)	0.66 (0.27–1.62)		
Mixed occupations	1.02 (1.00–1.04)	Carpenters	1.04 (0.80–1.35)	Farmers (61)	0.94 (0.77–1.14)		
Public safety workers–firefighters	1.00 (0.90–1.12)	Cleaners	1.25 (0.86–1.80)	Production supervisors and general foremen (70)	1.1 (0.89–1.37)		
Other construction workers	0.98 (0.96–1.00)	Clerks	0.92 (0.83–1.02)	Metal processers (72)	1.14 (0.93–1.39)		
Engine and motor operators	0.91 (0.55–1.51)	Cooks	1.13 (0.93–1.38)	Sawyers, plywood makers and related wood-processing workers (732)	1.51 (0.91–2.48)		
Other workers	0.48 (0.13–1.80)	Electricians	1.04 (0.87–1.24)	Chemical processors (74)	1.09 (0.82–1.45)		
		Firefighters	1.26 (0.76–2.09)	Petroleum-refining workers (745)	0.52 (0.10–2.69)		
		Fishery workers	0.81 (0.62–1.06)	Textile workers (75)	1.05 (0.81–1.36)		
		Food processors	1.03 (0.90–1.18)	Butchers and meat preparers (773)	0.99 (0.70–1.41)		
		Forestry workers	1.00 (0.80–1.24)	Food preservers (774)	1.07 (0.34–3.32)		
		Freight handlers	1.10 (0.94–1.28)	Dairy product processers (775)	1.26 (0.61–2.60)		
		Gardeners	1.19 (0.84–1.70)	Tailors, dressmakers, sewers, upholsterers (79)	1.07 (0.77–1.49)		
		Hand packers	0.99 (0.70–1.41)	Leather workers (801–803)	1.31 (0.89–1.94)		
		Health professionals	1.07 (0.87–1.31)	Motor-vehicle mechanics (843)	1.16 (0.90–1.50)		
		Managers	1.39 (0.95–2.03)	Electrical fitters and related electrical and electronics workers (85)	1.1 (0.91–1.34)		
		Nurses	0.90 (0.44–1.85)	Plumbers (871)	0.98 (0.69–1.39)		
		Plumbers	1.23 (0.97–1.56)	Welders (872)	1.22 (0.91–1.63)		
		Police officers and guards	1.03 (0.78–1.36)	Rubber workers (901, 902)	1.18 (0.84–1.67)		
		Printers	1.10 (0.87–1.40)	Paper and paperboard product makers (91)	0.96 (0.41–2.24)		
		Protective service occupations	0.94 (0.84–1.05)	Painters (93)	1.17 (0.91–1.50)		
		Sheet metal workers	1.09 (0.57–2.07)	Asbestos cement product maker (94330)	0.64 (0.16–2.49)		
		Tailors	1.33 (0.93–1.90)	Reinforced concreters, cement finishers and terrazzo workers (952)	1.17 (0.86–1.59)		
		Teaching professionals	0.98 (0.70–1.37)	Roofers (953)	0.72 (0.36–1.43)		
		Tool-makers	1.17 (0.86–1.59)	Carpenters, joiners and parquetry workers (954)	1.04 (0.81–1.34)		
		Welders	1.04 (0.88–1.23)	Plasterers (955)	1.69 (0.84–3.41)		
				Insulators (956)	1.67 (0.61–4.59)		
				Construction workers NEC (959)	0.88 (0.68–1.16)		
				Stationary engine and related equipment operators (96)	1.07 (0.72–1.57)		
				Railway engine-drivers and firemen (983)	1.41 (0.87–2.28)		
				Railway brakemen, signalmen and shunters (984)	1.43 (0.77–2.63)		
				Motor vehicle drivers (985)	1.14 (0.97–1.33)		
				Industries (ISIC code^$^) [184]			
				Salt mining (2903)	4.41 (1.43–13.6)		
				Manufacture of carpets and rugs (3214)	4.07 (1.44–11.5)		
				Manufacture of paints, varnishes and lacquers (3521)	2.94 (1.48–5.84)		
				Manufacture of plastic products NEC (356)	1.79 (1.06–3.00)		
				Manufacture of industrial chemicals (351)	1.58 (1.07–2.33)		
				Education services (931)	1.47 (1.06–2.05)		

* ISCO (International Standard Classification of Occupations); ^$^ ISIC (International Standard Industrial Classification)
